# One Step before Synthesis: Structure–Property–Condition Relationship Models to Sustainable Design of Efficient TiO_2_-Based Multicomponent Nanomaterials

**DOI:** 10.3390/ijms232113196

**Published:** 2022-10-30

**Authors:** Alicja Mikolajczyk, Dawid Falkowski

**Affiliations:** 1 Laboratory of Environmental Chemoinformatics, Faculty of Chemistry, University of Gdansk, Wita Stwosza 63, 80-308 Gdansk, Poland; 2 QSAR Lab, Trzy Lipy 3, 80-172 Gdansk, Poland

**Keywords:** multicomponent nanomaterials characterization, advanced materials design, nanophotocatalysts, sustainability-by-design, QSPR model

## Abstract

To control the photocatalytic activity, it is essential to consider several parameters affecting the structure of ordered multicomponent TiO_2_-based photocatalytic nanotubes. The lack of systematic knowledge about the relationship between structure, property, and preparation parameters may be provided by applying a machine learning (ML) methodology and predictive models based on the quantitative structure-property-condition relationship (QSPCR). In the present study, for the first time, the quantitative mapping of preparation parameters, morphology, and photocatalytic activity of 136 TiO_2_ NTs doped with metal and non-metal nanoparticles synthesized with the one-step anodization method has been investigated via linear and nonlinear ML methods. Moreover, the developed QSPCR model, for the first time, provides systematic knowledge supporting the design of effective TiO_2_-based nanotubes by proper structure manipulation. The proposed computer-aided methodology reduces cost and speeds up the process (optimize) of efficient photocatalysts’ design at the earliest possible stage (before synthesis) in line with the sustainability-by-design strategy.

## 1. Introduction

Over the past few years, finding new ways to cut CO_2_ emissions (at least 40% by 2030, compared with 1990) and increasing renewable energy consumption, while maintaining economic growth, - are both industrial and EU key targets. One of the accessible approaches, simultaneoufsly allowing the utilization of CO_2_, is the utilization of the energy from light (from UV to NIR) via heterogeneous photocatalysis [1], photoelectrochemical reduction [2], or photocatalytic process [3] with the use of TiO_2_ nanostructures that convert CO_2_ into stable, valuable chemicals (fuels or raw materials). TiO_2_ nanoparticles (NPs) are the most widely investigated material, considered promising and efficient photocatalysts. TiO_2_ nanoparticles are characterized by high photoactivity, relatively low cost, low toxicity, and good chemical and thermal stability. However, TiO_2_ has low quantum efficiency under visible light, resulting from the fast recombination rate of photogenerated e^-^/h^+^ pairs [4]. Thus, current systems based on TiO_2_ NPs are still too expensive and inefficient for commercial deployment.

Various strategies for modifying TiO_2_ in heterogeneous photocatalysis have been applied to overcome the abovementioned drawbacks. The design process of new efficient TiO_2_-based systems has been focused on the enhancement of its intrinsic properties by metal and non-metal doping (e.g., TiO_2_ combining with noble/rare metal nanoparticles or narrow band gap semiconductor particles [5,6]), as well as metal chalcogenides, graphene-based composites [7], oxygen type perovskites [8], metal-organic frameworks (MOFs) [9], g-C_3_N_4_ [10], and conducting polymers.

Among these strategies, the preparation of well-organized TiO_2_ nanoparticles in the form of nanotubes with metal and non-metal doping metals in the anodic oxidation process appears to be a highly efficient method to tune the response of the semiconductor to the visible light region and enhance its photocatalytic properties. The anodic oxidation process occurs in electrolytes with double- or triple-electrode systems. Pure or doped titania foil is used as an electrode. During the anodization process, layers of organized titanium dioxide nanotubes are obtained [5]. Many heterogeneous NP-base photocatalysts have been described in the literature, for example, different types or concentrations of an electrolyte, such as ionic liquids [11,12,13], or urea as nitrogen precursor [14], titanium foil used as working electrode interlaced with modifiers [15,16] or decorated by Pt nanoparticles and Bi_2_S_3_ quantum dots [17].

Unfortunately, the main challenge in the new TiO_2_-based nanostructure design is that there are thousands of possible combinations of nanoparticles’ structural features (e.g., sizes, surface area, metal, non-metal doping, etc.). Considering that it would be a time-consuming, expensive, and complicated experimental study, it is irrational to experimentally synthesize and test all possible structure combinations to find the most effective photocatalysts. In the literature, there is a lack of systematic knowledge of how the experimental conditions influence changes in heterogeneous nanostructure and how the change in the nanostructure appears to tune the response of the semiconductor to the visible light region to enhance its photocatalytic properties. So far, the development of heterogeneous nanoparticles was based on subjective experts’ expectations and very narrow investigations, rather than systematic studies of a wide space of possible solutions. In effect, research is conducted based on subjective knowledge, which leads to synthesizing each combination of modifiers and then measuring their activity. This way of synthesis is ineffective and time-consuming. This challenge can be faced by applying appropriate computational methods (virtual design) based on combined machine learning methods and virtual screening methodology. Quantitative mapping of the relationships between structure combination (metal and non-metal doping) of TiO_2_ NTs and experimental conditions enables researchers to thoroughly understand and control the photocatalytic properties, which are essential for future developments in this research area, as well as efficient systems design. However, because of the complex structure of heterogeneous nanomaterials, developing these correlations will require extensive studies, high-throughput techniques, and new strategies. One of the most promising approaches that may fulfill the above described lack of data is Quantitative Structure-Property Relationship Modeling for advanced and multicomponent nanomaterials. QSPR methods for the multicomponent nanomaterials (so-called nano-QSAR_mix_) were introduced for the first time in 2016 by Mikolajczyk et al. [18]. The nano-QSAR_mix_ allows for predicting the impact of structural modifications on the photocatalytic activity of newly designed photocatalysts. It facilitates the identification of more meaningful relationships between the experimental and structural identity of TiO_2_ NTs and their photocatalytic outcome under visible light. Recently, Mikolajczyk et al. [18,19] established how to adjust the QSPR methodology to predict the photocatalytic activity of newly designed TiO_2_-based nanostructures modified with noble metals. This method has been successfully applied in predicting the structure-photocatalytic activity relationship modeling of different TiO_2_-based photocatalysts. However, up to today, there has been no study that provides systematic knowledge about the quantitative relationship between experimental conditions, structure modification, and photocatalytic activity of newly designed TiO_2_-based photocatalysts.

The presented research aimed to invent a computational screening methodology for studying the role of experimental conditions and structural features of well-organized doped- and modified-TiO_2_ nanotubes (NTs) on its photocatalytic efficiency at the early stage of advanced photocatalyst design (before synthesis). In the first step, a database of 136 TiO_2_ well-organized doped- and modified-TiO_2_ nanotubes (NTs) was collected. Then, linear and nonlinear ML methods were developed to provide systematic knowledge about the relationship between experimental conditions, nanostructure, and photocatalytic properties of newly designed, well-organized doped- and modified-TiO_2_ nanotubes. Then, ML-based models and predictive nano-QSAR_mix_ were developed for the first time to provide systematic knowledge answering the question of how to control the photocatalytic properties of newly designed TiO_2_-based nanotubes by proper structure manipulation and how to manipulate the structure by experimental conditions modification. As an effect of the virtualization of the design process, the experimental procedure would become much faster, much more efficient in terms of the number of considered solutions, and less expensive. Thus, improving computer-aided methods of designing new materials will contribute to optimizing the design process while reducing costs and time intended for the new materials development process.

## 2. Results and Discussion

### 2.1. Use of HCA Analysis to Compare the Similarity of Designed TiO_2_-Based NTs

**Figure 1:**
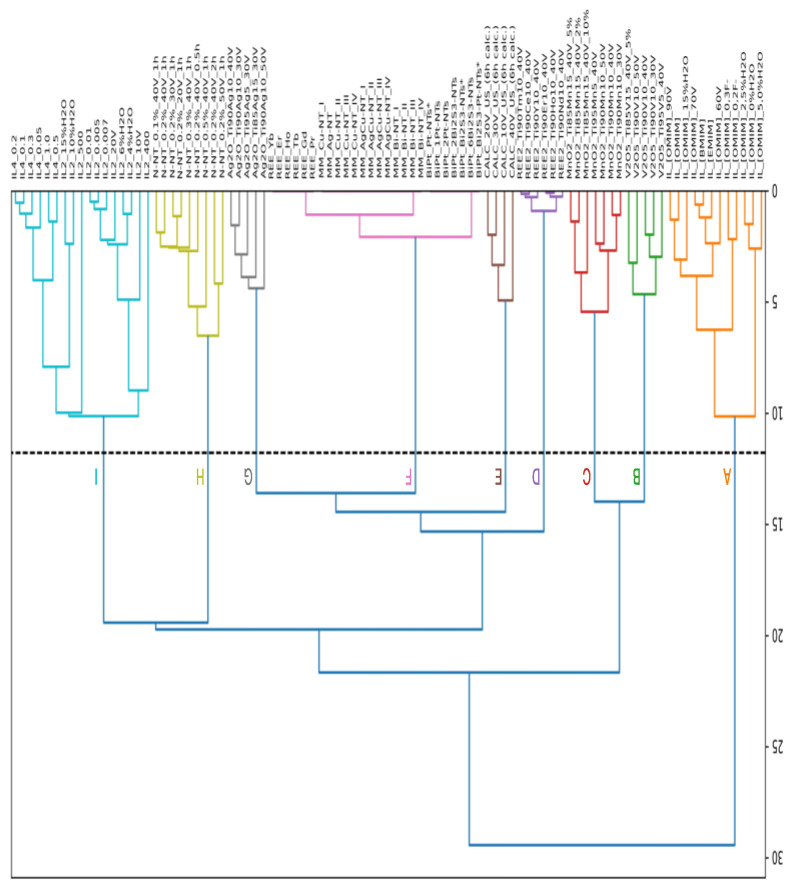
The schematically represented Hierarchical Cluster Analysis (HCA) for selecting groups of similar TiO_2_-based NTs active under VIS and UV-VIS light.

The data from 113 TiO_2_-based NTs modified by metal and non-metal nanoparticles derived from the literature were collected into one matrix (Table S1) [11,12,13,14,15,16]. The designed TiO_2_-based NTs were characterized by experimental conditions, structural modification of TiO_2_-based NTs, and photocatalytic properties (Table S2). To compare the influence of proposed metal and non-metal modification on the structure of TiO_2_-based NTs, the Hierarchical Clustering Analysis (HCA) was performed (Figure 1). The HCA was used to explore the distribution of the selected TiO_2_-based NTs in the space of structural similarity. The HCA indicates that TiO_2_-based NTs were grouped into nine clusters of self-organized TiO_2_ nanotube arrays interlaced with metal and non-metals particles. Each cluster differs from the others in terms of the presence of metal and non-metal nanoparticles that belong to a given group, such as (A) well-ordered nanotubes in the presence of different types of ionic liquids (TiO_2_/ILs) [13], (B) hierarchical V_2_O_5_–TiO_2_ NPs obtained from Ti-V alloys (TiO_2_/V_2_O_5_) [20], (C) TiO_2_–MnO_2_ NPs obtained from alloys (TiO_2_/MnO_2_), (D) rare-earth metals-modified TiO_2_ NTs (TiO_2_/REE) [16], (E) calcinated titanium dioxide nanotubes [21], (F) monometallic-modified TiO_2_ (Er, Yb, Ho, Tb, Gd, Pr, Cu, Ag, Bi, Pt), bimetallic modified (AgCu) and BiS quantum dots modified TiO_2_ NTs [17,22,23], (G) self-organized TiO_2_ interlaced with silver nanoparticles (TiO_2_/Ag_2_O) [15], (H) nitrogen doped TiO_2_ NTs (TiO_2_/N) [14], and (I) nanotubes modified with the presence of ionic liquids in different conditions (TiO_2_/ILs) [11,12]. HCA analysis indicates that the presence of metal and non-metal elements that incorporate into the TiO_2_ structure during nanotube formation is crucial for controlling the morphological features of the designed TiO_2_-based NTs. These findings are in agreement with the literature data [14].

### 2.2. How to Control the Morphology of Designed TiO_2_-Based NTs? Virtual Screening of Structure—Experimental Condition Relationship

The experimental study [16] indicates that photocatalytic degradation of pollutants under visible and light is mainly related to photogenerated electrons and superoxide radicals. Moreover, the photoelectrochemical tests [16,24,25] proved that the presence of REE-modification of TiO_2_-based NTs had an influence on increased photocurrent. The study provided by Nevárez-Martínez et al. [26] indicates that MnO_2_ species may absorb visible light irradiation, which is crucial to promote the enhancement of the charge transfer rate. Thus, the transfer of photoexcited electrons (generated in TiO_2_ NTs) within MnO_2_ under visible light may improve the photocatalytic activity of TiO_2_/MnO_2_ systems. Similar results were described by Mazierski and al. [15] for TiO_2_/Ag_2_O nanotubes. The authors [15] proved that photocatalytic efficiency is related to the optimal content of Ag_2_O and Ag NPs, which are crucial for the number of recombination centers under UV-VIS light. While the activity under VIS light is correlated with increasing Ag_2_O and Ag^0^ content in the TiO_2_/Ag_2_O-based NTs, due to the utilization of a higher amount of incident photons.

The results described in the presented HCA analysis are in agreement with the experimental evidence [15,16,24,25,26]. HCA analysis (Figure 1) confirms that doping with metal or non-metal elements is crucial for controlling the morphological features of TiO_2_-based NTs and, consequently, their photocatalytic activity. While the morphology, i.e., length and wall thickness of TiO_2_ nanotubes, top-opened or clogged, and wall smoothness is controlled by experimental conditions formed throughout the one-step anodization process. In this context, the Principal Component Analysis (PCA) was developed to explore the distribution of the TiO_2_-based NTs photocatalysts in the space of the experimental conditions and the structural features of TiO_2_-based NTs.

The PCA analysis was performed for the groups with a defined endpoint expressed as phenol photodegradation rate (λ, μmol·dm^−3^·min^−1^) under VIS and UV-VIS light (Table S2). Thus, the final PCA analysis was performed for clusters selected from HCA (Figure 1), such as: (A) well-ordered nanotubes in the presence of different types of ionic liquids (TiO_2_/ILs) [13], (D) rare-earth metals-modified TiO_2_ NTs (TiO_2_/REE) [16], (E) calcinated titanium dioxide nanotubes [21], (F) monometallic-modified TiO_2_ (Er, Yb, Ho, Tb, Gd, Pr, Cu, Ag, Bi, Pt), bimetallic modified (AgCu), and BiS quantum dots modified TiO_2_ NTs [17,22,23], (G) self-organized TiO_2_ interlaced with silver nanoparticles (TiO_2_/Ag_2_O) [15], (H) nitrogen-doped TiO_2_ NTs (TiO_2_/N) [14], and nanotubes modified with the presence of ionic liquids in different conditions (TiO_2_/ILs) [11,12] (Figure 1). Thus, the final PCA analysis was prepared for 32 TiO_2_-based NTs.

**Figure 2:**
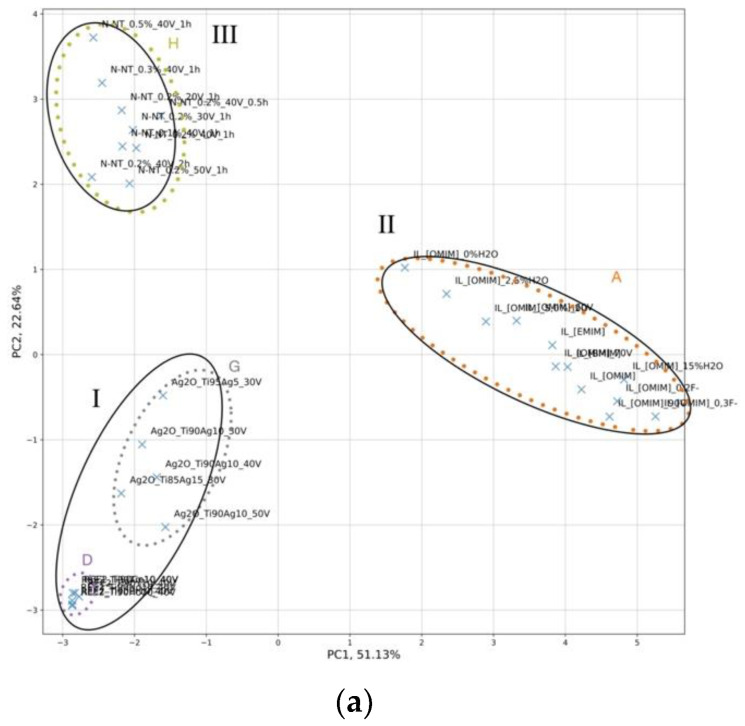

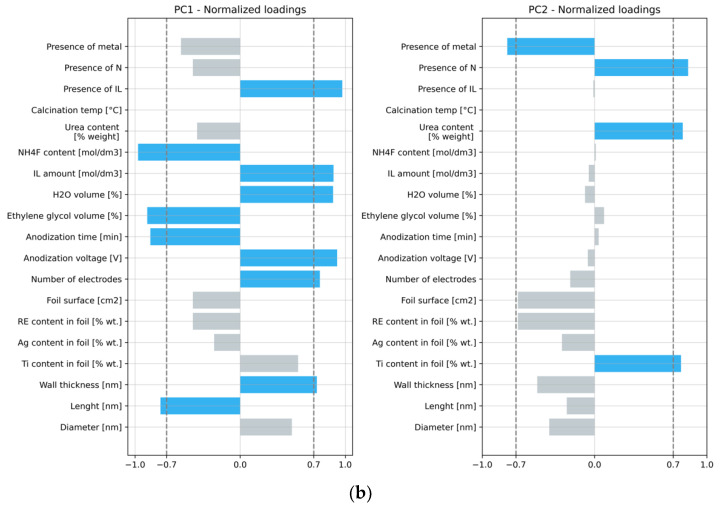
(**a**) Score plots from the two PC analyses performed for 32 well-ordered TiO_2_-based NTs in the space of descriptors that represent the experimental conditions and structural features of TiO_2_-based NTs; (**b**) Correlation coefficients between a selected descriptor and either PC1 or PC2 (loadings values). Color codes: Values marked in blue are significant in particular PCs and determine the physical interpretation of these PCs.

The results of the constructed PC map in the space of structural features and experimental conditions are displayed in Figure 2. The first two principal components (PC1 and PC2) explained 73.8% (51.1% + 22.6%) of the total variance in the data. The physical interpretation of a given PC can be assigned based on the contributions of the original descriptors to that PC (loadings values) schematically represented in Figure 2b.

**Figure 3:**
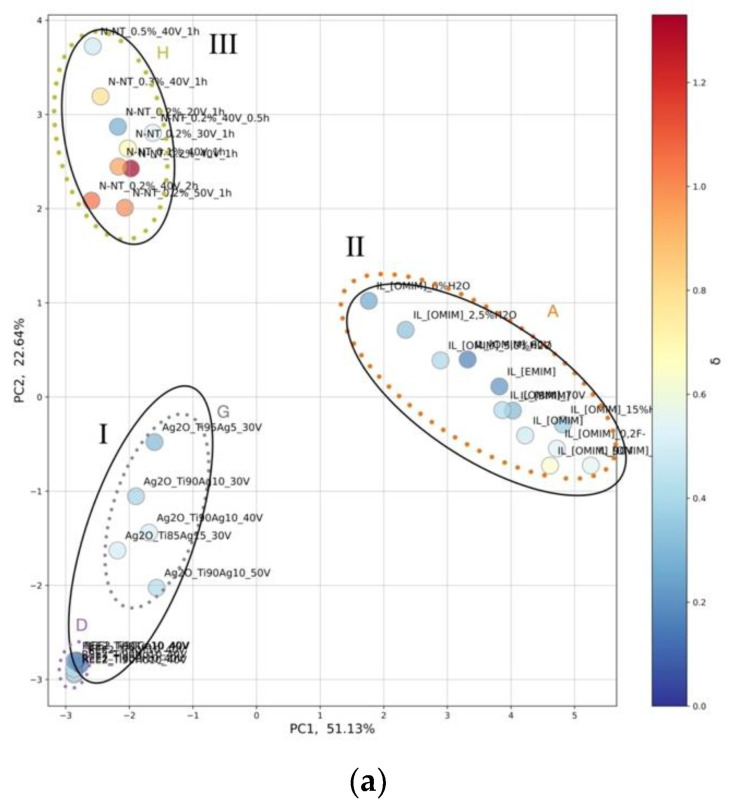

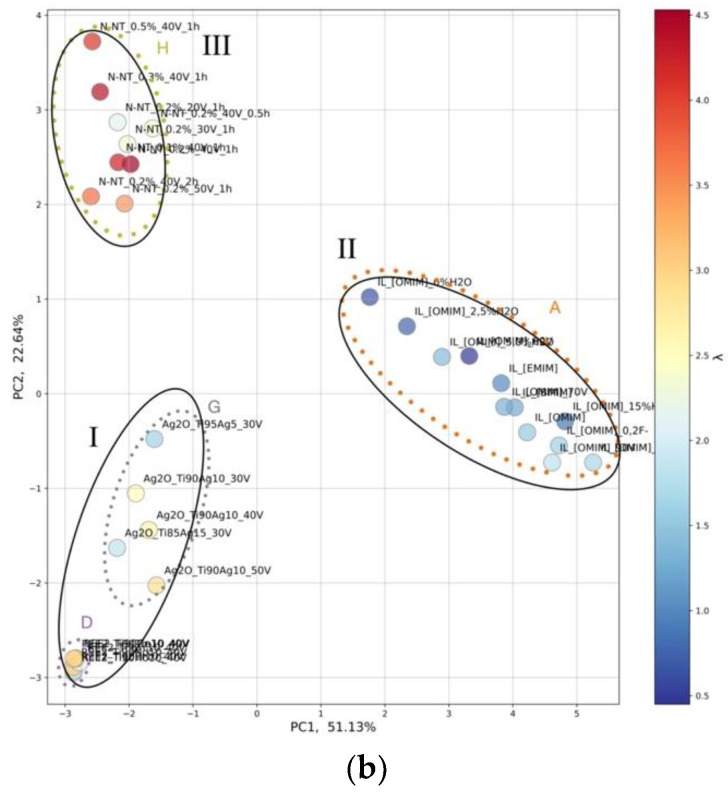
Score plots from the two PC analyses performed for 32 well-ordered TiO_2_-based NTs in the space of descriptors that represent experimental conditions and structural features of the investigated 32 well-ordered TiO_2_-based NTs for phenol photodegradation rate (λ, μmol·dm-3·min-1) under (**a**) VIS and (**b**) UV-VIS light. Color codes: Values marked in blue and red determine the photocatalytic activity of selected samples.

To correlate the dependency between experimental conditions, morphology, and photocatalytic activity, the results obtained from PCA were transferred into the color range, in which the ranges corresponded to the values of the photocatalytic activity expressed as phenol photodegradation rate (λ, μmol·dm^−3^·min^−1^) under VIS and UV-VIS light, respectively (Figure 3). In this way, we tried identifying systematic patterns in the data that might suggest which structural features and experimental conditions are mainly responsible for the efficiency of the designed 32 well-ordered TiO_2_-based NTs.

Along with PC1, there are two main clusters. The first cluster contains Groups I and III, which includes groups D, G, and H from HCA (Figure 1, Table S2), such as rare-earth metals-modified TiO_2_ NTs (TiO_2_/REE), self-organized TiO_2_ interlaced with silver nanoparticles (TiO_2_/Ag_2_O), and nitrogen-doped TiO_2_ NTs (TiO_2_/N), and (I) nanotubes modified with the presence of ionic liquids in different conditions (TiO_2_/ILs). The second cluster contains Group II, which includes group A from HCA (Figure 1, Table S2) represented by well-ordered nanotubes in the presence of different types of ionic liquids (TiO_2_/ILs), Table S2. Along with PC2, there are three main groups. Group I contains rare-earth metals-modified TiO_2_ NTs (TiO_2_/REE), self-organized TiO_2_ interlaced with silver nanoparticles (TiO_2_/Ag_2_O), Group II contains well-ordered nanotubes in the presence of different types of ionic liquids (TiO_2_/ILs). While Group III contains nitrogen-doped TiO_2_ NTs (TiO_2_/N).

**Figure 4:**
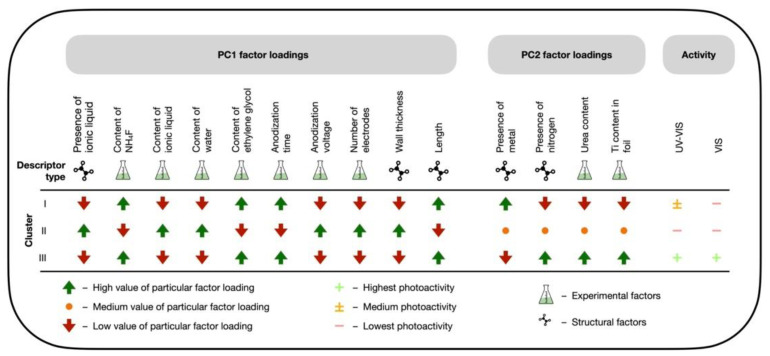
The graphical interpretation of the finding from PCA analysis that presents the relationship between experimental conditions, structural features, and photocatalytic efficiency.

The PCA design map (Figure 4) that shows the correlation between loading values conducted within PC1 and PC2 and photocatalytic activity transferred into the color range was prepared in order to understand the relationship between experimental conditions and structure features that may be fundamental in the future design of new and efficient TiO_2_-based NTs.

As it can be seen from Figure 4, the activity under VIS and UV-VIS light are correlated mainly with length, wall thickness, and the presence of non-metal elements. The highest activity under both VIS and UV-VIS light is obtained for non-metal-doped TiO_2_ nanotubes. The activity increased with the length of TiO_2_ NTs and decreased wall thickness of TiO_2_ NTs. Both parameters depend on anodization time, anodization voltage, and the number of electrodes (Figure 3a,b). This observation complies with results obtained by experimental methods [11]. The PCA analysis indicates that a higher length of TiO_2_ NTs and lower wall thickness of TiO_2_ NTs are obtained with longer anodization time, lower voltage, and, consequently, a lower number of electrodes.

Moreover, the synthesis should be controlled by the amount of water and Ti content in the foil. The anodization process should be carried out in the electrolyte with a higher amount of ethylene glycol and a lower amount of water, using NH_4_F and different weight percentages of urea (as nitrogen precursor). These findings agree with the literature data [11,12,13,14,15,16]. For example, Nischk and al. [22] indicated that the photocatalytic activity, as well as the charge carrier recombination rate, depend on nitrogen concentration and process parameters, such as voltage and anodization time. The authors [22] indicated that the dimensions of nanotubes could be easily controlled within a wide range by changing the applied voltage and anodization time. Similar results were obtained by Pancielejko et al. [11,12]. Authors indicated that the photocatalytic activity and the charge carrier recombination rate may depend on nitrogen concentration. The TiO_2_ NTs in this group were grown in an ethylene glycol-based electrolyte that contained more NH_4_F than other samples [14,15,16]. The NTs in this group were grown at lower anodization voltage and longer anodization time using Ti sheets in an organic electrolyte containing specified amounts of urea as nitrogen precursor. The most active samples are characterized by higher length and lower wall thickness of TiO_2_ NTs compared with groups I and II.

Interestingly, Mazierski et al. [14] concluded that there are no significant differences in nitrogen-doped TiO_2_-based NTs’ length under higher voltages and longer anodization time. However, there is a significant difference in length as compared to nanotubes obtained with lower voltages and shorter anodization time. This conclusion indicates that the virtual screening methodology of a wider group of TiO_2_-based NTs may bring new insight into the relationship between experimental conditions, structural features, and photocatalytic activity that is crucial for the design process of new TiO_2_-based NTs. For example, [16] indicates that nanotube dimensions for rare-earth metals-modified TiO_2_ nanotubes strongly depend on the anodization potential, including anodization time and voltage.

### 2.3. The Quantitative Relationship between Structure, Synthesis Condition, and Photocatalytic Activity of TiO2-Based NTs: Predictive Nano-QSPCRmix Model Development

To develop linear correlations between the determined structural, experimental descriptors (x), and the measured photocatalytic activity endpoints (*y*), multiple linear regressions (MLR) with a genetic algorithm (GA) were used to model y as a function of x, y = f(x).

**Figure 5:**
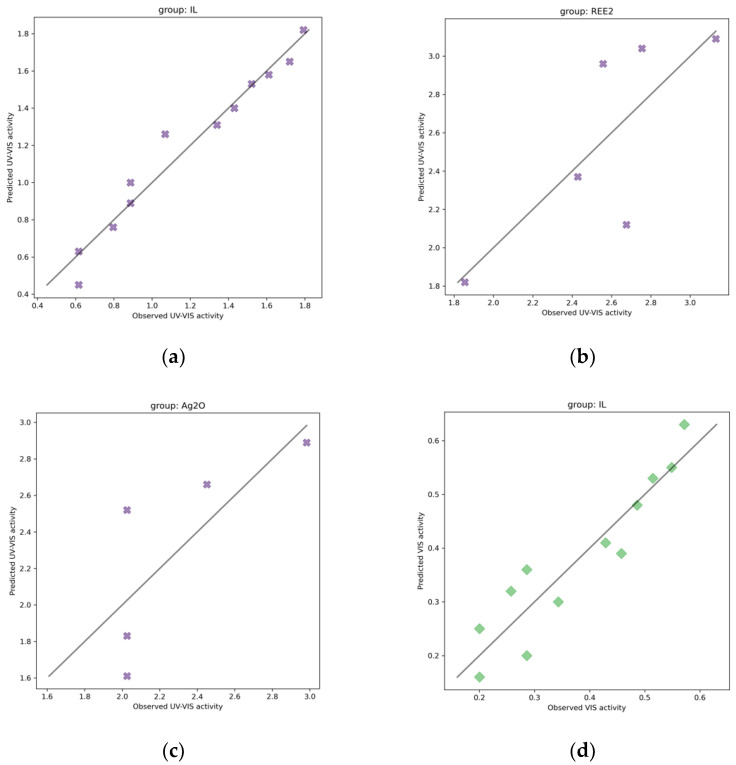

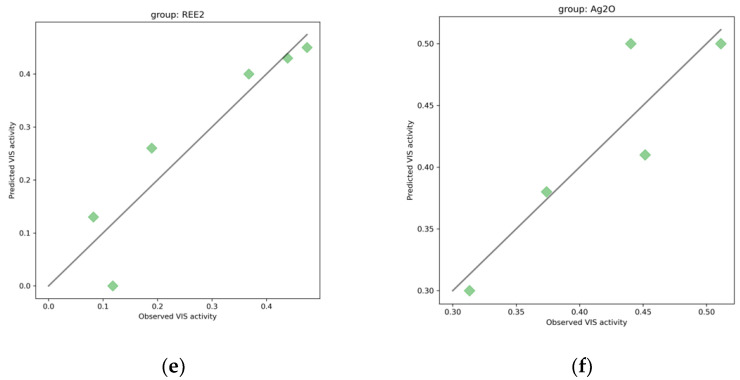
The MLR models for self-organized TiO_2_ active under UV-VIS for: (**a**) well-ordered nanotubes in the presence of different types of ionic liquids (TiO_2_/ILs), (**b**) rare-earth metals-modified TiO_2_ NTs (TiO_2_/REE), and (**c**) interlaced with silver nanoparticles (TiO_2_/Ag_2_O); active under VIS light (**d**) well-ordered nanotubes in the presence of different types of ionic liquids (TiO_2_/ILs), (**e**) rare-earth metals-modified TiO_2_ NTs (TiO_2_/REE), (**f**) interlaced with silver nanoparticles (TiO_2_/Ag_2_O). Particular models’ equations and statistics are shown in Table S3.

Table 1 and Figure 5 compare the performance of the constructed linear regression models for predicting photocatalytic activity under VIS and UV-VIS light.

The MLR-GA models showed high accuracy, deduced by their acceptable R^2^, CCC and RMSE_C_, and MAE_C_. Interestingly, each MLR-GA model includes one descriptor that corresponds to the structure of TiO_2_-based NTs, such as length, Ti^3+^ ion content (based on XPS analysis) content, and total titanium contain (∑ Ti [% at.]). The MLR-GA indicates that photocatalytic activity under UV-VIS light increases with length, diameter, and Ti^3+^ ion content. While PCA analysis proved that these morphology features might be easily controlled by experimental conditions, i.e., Ti content in foil (higher), anodization time (higher), with higher amount of ethylene glycol and a lower amount of water in the electrolyte (Figure 4). These findings agree with the results present in the literature [14]. In the paper [14], authors proved that crystallite size, NTs length, and Ti^3+^ state content are critical to creating hydroxyl radicals. The presence of a Ti^3+^ state can reduce oxygen dissolved in water and produce superoxide radicals, while positive holes may be involved in the generation of •OH radicals that affect photocatalytic activity.

**Figure 6:**
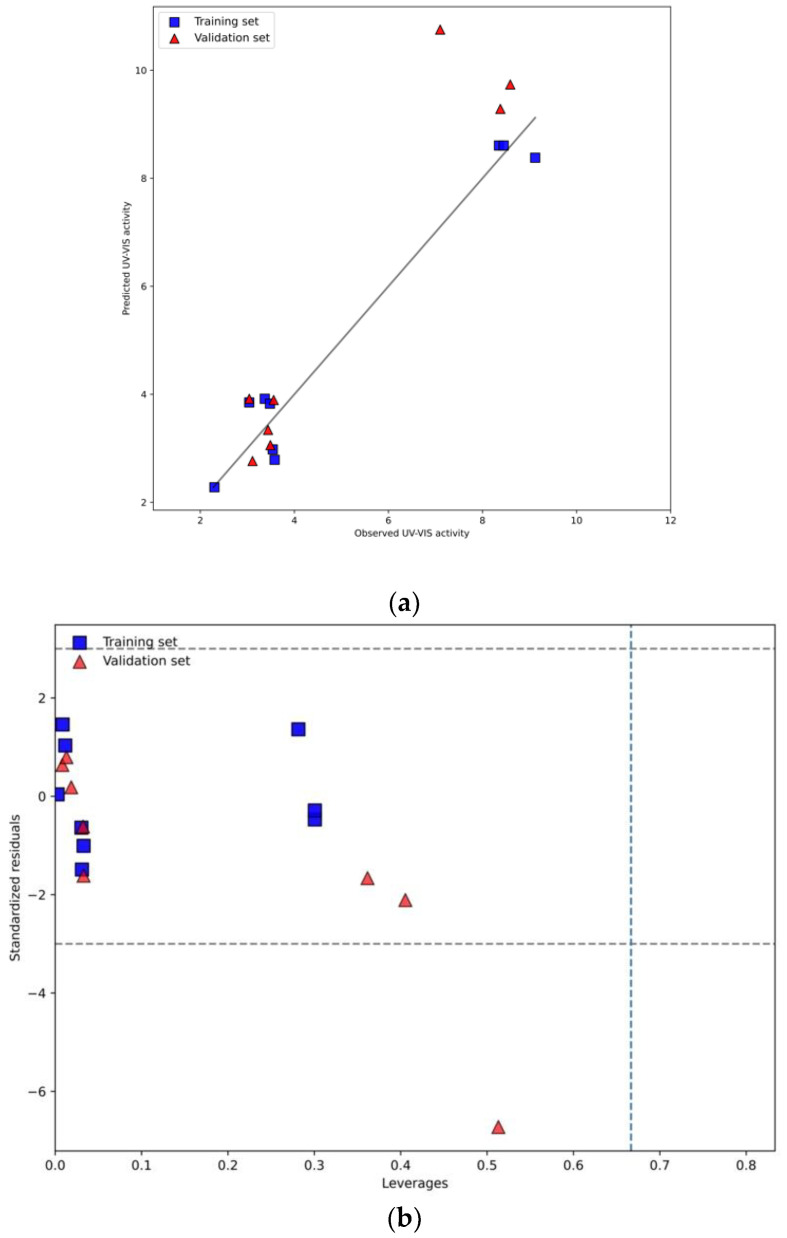
(**a**) Observed vs. predicted diagram for developed MLR-GA (**b**) Applicability domain represented by Williams plot.

Finally, the most representative dataset from HCA (Group I, see Figure 1) that contains 17 well-ordered nanotubes was used for the predictive nano-QSPCR_mix_ model development. The group is characterized by nanotubes modified with the presence of ionic liquids in different conditions (voltage, amount of ionic liquid, and water) (TiO_2_/ILs) [11,12]. The model (Equation (2)) was validated following recommendations of the Organization for Economic Co-operation and Development (OECD) [27]. Goodness-of-fit was assessed by the determination coefficient (R^2^), and the root means square error of calibration (RMSE_C_) was based on the prediction for the training set. The model’s robustness (stability) was verified by internal validation using the cross-validation leave-one-out algorithm (for MLR models). The robustness was expressed by Q_LOO_^2^; accordingly, root means square of cross-validation (RMSE_CV_) also was calculated. Predictive ability of all models was assessed by the external validation coefficient (R_EXT_^2^), the root means square error of prediction (RMSE_P_), and the Concordance Correlation Coefficient (CCC). The applicability domain (AD) was also developed (Figure 6b).

The developed for first-time nano-QSPCR_mix_ model is described by the following Equation (2) and utilizes a descriptor that represents a length of TiO_2_ NTs.

(1) $$\lambda_{UV–VIS}\;=\;1.60+0.0011*Lenght\;\left[nm\right]$$

The developed nano-QSPCR_mix_ model is characterized by the following statistical characteristics: R^2^ = 0.96, RMSE_C_ = 0.556, R_adj_^2^ = 0.95, R^2^–R_adj_^2^ = 0.01, CCC_tr_ = 0.98, MAE_tr_ = 0.47, Q^2^_LOO_ = 0.93, RMSE_CV_ = 0.69, R^2^–Q_LOO_^2^ = 0.03, CCC_cv_ = 0.96, MAE_cv_ = 0.60, Q^2^_F1_ = 0.61, Q^2^_F2_ = 0.61, Q^2^_F3_ = 0.69, R_EXT_^2^ = 0.90, RMSE_EXT_ = 1.45, CCC_ext_ = 0.87, MAE_EXT_ = 0.97, F = 152.08.

Observed-predicted plots and applicability domain areas of the nano-QSAR_mix_ models for photocatalytic activity are presented in Figure 6.

The finally developed nano-QSPCR_mix_ model (Equation (2)) explains 96% of the variability of observed photocatalytic activity under UV-VIS light (λ_UV–VIS_) of the investigated ionic liquids-modified titanium oxide nanotubes samples. The model Equation (2) indicates that there is a linear correlation between photocatalytic activity and the length of TiO_2_ NTs. Strong linear correlations between the observed and predicted values of (λ_UV–VIS_), graphically presented in Figure 6a, proved the good fit and high predictive ability of both models. Additionally, the plot of standardized cross-validated residuals versus leverages (Williams plot) confirm that all training and validation compounds are located within the applicability domain area, i.e., the area defined by structural similarity of the nanoparticles, where the predictions are reliable (Figure 6b). Interpretation of the descriptor (i.e., a length of TiO_2_ NTs) brings significant insight into the current knowledge on structural factors that are likely to affect the photocatalytic activity (λ_UV–VIS_) of the studied nanoparticles. The model (Equation (2)) indicates that photocatalytic activity increases with the length of TiO_2_ NTs (standardized coefficient: +0.0011). The developed nano-QSPCR_mix_ model quantifies the influence of the structural features (the length) of TiO_2_ NTs on the modeled photocatalytic activity (λ_UV–VIS_) which is of high importance for designing an efficient photocatalyst. The developed predictive nano-QSPCR_mix_ model is the first step in developing an ML-based framework that may significantly support the experimental design of novel photocatalysts based on TiO_2_ NTs.

The results obtained in our study regarding the influence of TiO_2_ NTs’ structure on photocatalytic activity (λ_UV–VIS_) are in agreement with the results presented in previously published papers by experimental groups [28,29,30]. Morphology and TiO_2_ structure modified by ionic liquids is one crucial parameter for increasing the efficiency of titanium nanotubes obtained in the electrochemical synthesis process. For example, according to results provided by Liu et al. [28], longer nanotubes increase the amount of TiO_2_ available for contact with pollutants where the photocatalytic reaction occurs. Furthermore, titanium nanotubes longer than 100 μm are characterized by higher mechanical stability, even for thick layers [29]. The longer length of nanotubes is associated with their higher survivability, which results in a longer period of operation of such a photocatalyst and consequently higher efficiency of the photocatalytic system during the entire service life. The tube shape, because of its higher aspect ratio, provides better optimized geometry for diffusion and low trapping and recombination kinetics of light-generated electron-hole pairs because the pairs don’t have to migrate between nanoparticles [30,31]. As a result, the inadvisable effect of electron hole recombination is limited. Paramasivam et. al. [32] indicate that the modification of dopants leads to reductions in the band gap; in effect, TiO_2_ nanotubes are excited in visible light. Also, the presence of ionic liquids increases the charge separation of photogenerated carriers at the photocatalyst surface, resulting in an increase in photocatalytic activity [33]. The ionic liquids’ presence in the reaction environment causes increased external dimensions of titania nanotubes, in comparison to nanotubes obtained without ionic liquids [11]. Additionally, authors [13] report that the presence of Ti^3+^ ions on the surface of TiO_2_ enhance photoactivity under UV-VIS light. Under UV-VIS irradiation, Ti^3+^ ions presence in the TiO_2_ matrix can facilitate electron-hole separation and promote the interfacial electron transfer process. This relationship was also proof by linear models for activity under UV-VIS light for REE-doped titania nanotubes shown in the Table 1. Moreover, self-organized titania nanotubes allow for use in the form in which they were made, unlike to titania nanotubes in powder form, which must be immobilized on a carrier by compacting or sintering or by being suspended in liquid [32].

## 3. Methodology

### 3.1. Dataset of Structure

The database of 113 self-organized TiO_2_ nanotubes (NTs) was fabricated via a one-step electrochemical anodization process described in previously published papers [11,12,13,14,15,16], Table S1. The data set includes TiO_2_ nanotubes formation in the presence of ionic liquids (ILs-NTs) [11,12,13], TiO_2_ nanotubes prepared at different calcination times [21], TiO_2_ nanotubes modified with monometallic (Cu, Bi) and bimetallic AgCu nanoparticles [22], well-organized TiO_2_ nanotubes modified by nitrogen (N-NT) [14], TiO_2_ NTs modified by rare metals (REE-NT) present in electrolyte [23] and in foil [16], self-organized nanotube Ag_2_O–TiO_2_ NTs arrays interlaced with silver nanoparticles [15], heterojunction-based ordered TiO_2_ nanotubes modified with platinum NPs, and bismuth sulfide quantum dots [17], TiO_2_–MnO_2_, and V_2_O_5_–TiO_2_ NPs obtained from Ti–V alloys [20,26].

### 3.2. Dataset of Endpoints

The photocatalytic activity of the obtained NTs was examined in a two-model reaction of the toluene and/or phenol degradation under VIS [13,14,15,16] and UV-VIS light [11,12,13,14,15,16], expressed as phenol photodegradation rate (λ, μmol·dm^−3^·min^−1^) under VIS and UV-VIS light. The details are described in previously published papers [11,12,13,14,15,16], Table S2.

### 3.3. Dataset of Descriptors

Our research has been focused on characteristics that determine unique properties of newly designed NTs, i.e., descriptors related to structure composition (so-called system independent properties), including the amount of chemicals used for surface-modification, surface-doping, high surface area, good adsorption ability, highly ordered array structure, open mesoporous nature, as well as descriptors that describe synthesis conditions (so-called system-dependent properties), such as anodization voltage and time, ultrasonic treatment, or calcination time. The preparation of samples and surface properties’ characterization is described in previously published papers [11,12,13,14,15,16,17,20,21,22,23,26].

### 3.4. Hierarchic Clustering Analysis (HCA)

The Hierarchical Clustering Analysis (HCA) is a grouping method that allows for arrangement of the object (i.e., TiO_2_-based NTs with different compositions) into tiered, ordered clusters that can be used to explore the data and visualize their underlying structure. All clustering methods are built on the concept of similarity: the greater the distance between objects, the lesser their similarity [34]. In our work, we performed HCA on TiO_2_ NTs with different compositions on the linear maps to provide information concerning their distribution among structure properties. In the case of the presented study, the distance between objects was defined by the Euclidean distance and Ward’s clustering methods using Python Scripts [35].

### 3.5. Principal Component Analysis (PCA)

Principal Component Analysis (PCA) is a method commonly used to reduce data complexity by creating a new set (from the original dataset) with uncorrelated vectors and analyzing similarities in studied structures [36]. In this method, new variables, called principal components (PCs), are developed as linear combinations of the original ones, where the first PC explains the largest possible amount of the variance in the original dataset. The second and next PC explains the largest possible variance unexplained by the previously used PC and so on. Finally, every object from the original dataset is described by a set of PCs instead of the original variables.

In the present study, the PCA method was adjusted to group the studied TiO_2_-based NTs with different compositions based on their structural similarity. Thus, to find the relationship between TiO_2_-based NTs structures and their potential photocatalytic activity, we have presented the structures of TiO_2_-based NTs in the space of two PCs (expressed as score plot) assigned with Malinowski’s rules (i.e., the contribution of descriptors was selected based on the normalized loadings higher than 0.7).

In the final step, the grouped object was classified and transferred into the color range, in which the ranges corresponded to the values of the photocatalytic activity expressed as phenol photodegradation rate (λ, μmol·dm^−3^·min^−1^) under VIS and UV-VIS light (Figure 3).

### 3.6. Nano-QSAR for Multicomponent Nanomaterials (Nano-QSARmix)

In the present study, QSARINS software has been used to develop Nano-QSAR_mix_ models. The model was developed based on the Multiple Linear Regression (MLR) technique and dataset that were split into two sets: training set (to be used to develop a Nano-QSAR_mix_ model) and validation set (to be used only for validating the model’s predictive ability) [27]. To perform a splitting, the nanoparticles were sorted along with the increasing values of UV-VIS activity (Table 2). Then, every second NP was included in the validation set (V), whereas the remaining NPs formed the training set (T). In MLR, the endpoint (***y_i_***) is described as the best combination of the most relevant auto-scaled descriptors used as independent variables (***x_1_, x_2_, …, x_n_***) (1):

(2) $$y_{i}\;=\;b_{0\;}+b_{1}x_{1}+b_{2}x_{2}+\ldots +b_{n}x_{n}\;$$

The correlation coefficient (R^2^) and the root mean square error of calibration (RMSEC) were used as the measures of goodness-of-fit for each developed model [18]. To verify the stability of the models (sensitivity on the composition of the selection of the training set), the cross-validated coefficient *Q*^2^_LOO_ (leave-one-out method) and root mean square error of cross-validation RMSECV were calculated [18]. In addition, the leverage approach and Williams plot were developed to assess applicability domain (AD) of the models. This was undertaken to verify the space defined by the structural similarity of nanoparticles and the values of UV-VIS activity, in which the model can make predictions with the most optimal reliability [27].

## 4. Conclusions

The effect of the experimental conditions in a one-step anodization process with the use of ML methods was studied to determine their influence on the morphology and on the photocatalytic properties of newly designed TiO_2_-based NTs. The linear and non-linear ML-based methodology, proposed here, for the first time, provides systematic knowledge about the relationship between experimental conditions, structure features, and photocatalytic activity under VIS and UV-VIS light. As an effect of integrated data-driven strategy and predictive nano-QSPCR_mix_ models, experimentalists will be able to identify and precisely control structural features of the TiO_2_ NTs by the proper manipulation of experimental conditions. This will allow the design and control of photocatalytic properties at the early stage of TiO_2_ NTs design (before synthesis). The proposed methodology may significantly support the experimental design of novel TiO_2_ NTs with desired properties. This may significantly speed up the whole designing process of a wider group of environmentally friendly TiO_2_-based nanomaterials in terms of the number of considered solutions, while reducing the costs and time of required experiments. We believe that the proposed study using computer-aided study is crucial for accelerating the design process of sustainable materials in line with the safe-and-sustainability by design (SSbD) strategy.

## Figures and Tables

**Table 1:** Summary of Linear models for 23 TiO_2_-based NTs.

Endpoint	Group Name	Group Size	Model Equation	Statistics				Ref. to Figure 5
				R2	RMSE_C_	MAE_C_	CCC	
UV-VIS	IL	12	y = 1.19 + 0.411 × Length	0.96	0.09	0.06	0.98	(a)
UV-VIS	REE2	6	y = 2.58 + 0.41 × Ti^3+^	0.61	0.31	0.23	0.76	(b)
UV-VIS	Ag_2_O	5	y = 2.42 + 0.60 × Diameter	0.58	0.32	0.28	0.74	(c)
VIS	IL	12	y = 0.382 + 0.13 × Length	0.87	0.05	0.04	0.93	(d)
VIS	REE2	6	y = 0.29 + 0.16 × Length	0.86	0.06	0.05	0.93	(e)
VIS	Ag_2_O	5	y = 0.40 − 0.06 × ∑ Ti [% at.]	0.80	0.03	0.03	0.89	(f)

**Table 2:** Descriptors selected for the model and the modeled endpoint values.

	Descriptor	UV-VIS Activity		
Sample	Length [nm]	Experimental	Predicted	Set ^1^
IL2_10 V	600	2.30	2.28	T
IL2_500	2050	3.04	3.92	V
IL2_400	1990	3.04	3.85	T
IL2_20 V	1030	3.11	2.76	V
IL2_0.01	2050	3.37	3.92	T
IL2_4%H_2_O	1540	3.44	3.34	V
IL2_0.005	1970	3.48	3.83	T
IL2_6%H_2_O	1290	3.49	3.06	V
IL2_10%H_2_O	1220	3.54	2.98	T
IL2_0.007	2030	3.56	3.89	V
IL2_15%H_2_O	1050	3.58	2.79	T
IL4_1.0	8100	7.10	10.75	V
IL4_0.1	6200	8.35	8.61	T
IL4_0.3	6800	8.38	9.28	V
IL4_0.2	6200	8.45	8.61	T
IL4_0.5	7200	8.59	9.74	V
IL4_0.05	6000	9.12	8.38	T

^1^ T–training set, V–validation (test) set.

## Data Availability

The data presented in this study are openly available online.

## Supplementary Materials

The following supporting information can be downloaded at: https://www.mdpi.com/article/10.3390/ijms232113196/s1. References [37,38] are cited in the Supplementary Materials.

## References

[1] White J.L., Baruch M.F., Pander J.E., Hu Y., Fortmeyer I.C., Park J.E., Zhang T., Liao K., Gu J., Yan Y. (2015). Light-Driven Heterogeneous Reduction of Carbon Dioxide: Photocatalysts and Photoelectrodes. Chem. Rev..

[2] Dalle K.E., Warnan J., Leung J.J., Reuillard B., Karmel I.S., Reisner E. (2019). Electro- and Solar-Driven Fuel Synthesis with First Row Transition Metal Complexes. Chem. Rev..

[3] Gonzales J.N., Matson M.M., Atsumi S. (2019). Nonphotosynthetic Biological CO_2_ Reduction. Biochemistry.

[4] Mazierski P., Mikolajczyk A., Bajorowicz B., Malankowska A., Zaleska-Medynska A., Nadolna J. (2018). The Role of Lanthanides in TiO_2_-Based Photocatalysis: A Review. Appl. Catal. B Environ..

[5] Zaleska-Medynska A. (2020). Metal Oxide-Based Photocatalysis.

[6] Li X., Yu J., Jaroniec M., Chen X. (2019). Cocatalysts for Selective Photoreduction of CO_2_ into Solar Fuels. Chem. Rev..

[7] Thakur K., Kandasubramanian B. (2019). Graphene and Graphene Oxide-Based Composites for Removal of Organic Pollutants: A Review. J. Chem. Eng. Data.

[8] Zeng S., Kar P., Thakur U.K., Shankar K. (2018). A Review on Photocatalytic CO_2_ Reduction Using Perovskite Oxide Nanomaterials. Nanotechnology.

[9] Wang Q., Astruc D. (2020). State of the Art and Prospects in Metal–Organic Framework (MOF)-Based and MOF-Derived Nanocatalysis. Chem. Rev..

[10] Shen M., Zhang L., Shi J. (2018). Converting CO_2_ into Fuels by Graphitic Carbon Nitride-Based Photocatalysts. Nanotechnology.

[11] Pancielejko A., Mazierski P., Lisowski W., Zaleska-Medynska A., Kosek K., Łuczak J. (2018). Facile Formation of Self-Organized TiO_2_ Nanotubes in Electrolyte Containing Ionic Liquid-Ethylammonium Nitrate and Their Remarkable Photocatalytic Properties. ACS Sustain. Chem. Eng..

[12] Pancielejko A., Mazierski P., Lisowski W., Zaleska-Medynska A., Łuczak J. (2019). Ordered TiO_2_ Nanotubes with Improved Photoactivity through Self-Organizing Anodization with the Addition of an Ionic Liquid: Effects of the Preparation Conditions. ACS Sustain. Chem. Eng..

[13] Mazierski P., Łuczak J., Lisowski W., Winiarski M.J., Klimczuk T., Zaleska-Medynska A. (2017). The ILs-Assisted Electrochemical Synthesis of TiO2 Nanotubes: The Effect of Ionic Liquids on Morphology and Photoactivity. Appl. Catal. B Environ..

[14] Mazierski P., Nischk M., Gołkowska M., Lisowski W., Gazda M., Winiarski M.J., Klimczuk T., Zaleska-Medynska A. (2016). Photocatalytic Activity of Nitrogen Doped TiO_2_ Nanotubes Prepared by Anodic Oxidation: The Effect of Applied Voltage, Anodization Time and Amount of Nitrogen Dopant. Appl. Catal. B Environ..

[15] Mazierski P., Malankowska A., Kobylański M., Diak M., Kozak M., Winiarski M.J., Klimczuk T., Lisowski W., Nowaczyk G., Zaleska-Medynska A. (2017). Photocatalytically Active TiO_2_/Ag_2_O Nanotube Arrays Interlaced with Silver Nanoparticles Obtained from the One-Step Anodic Oxidation of Ti–Ag Alloys. ACS Catal..

[16] Parnicka P., Mazierski P., Lisowski W., Klimczuk T., Nadolna J., Zaleska-Medynska A. (2019). A New Simple Approach to Prepare Rare-Earth Metals-Modified TiO_2_ Nanotube Arrays Photoactive under Visible Light: Surface Properties and Mechanism Investigation. Results Phys..

[17] Mazierski P., Nadolna J., Nowaczyk G., Lisowski W., Winiarski M.J., Klimczuk T., Kobylański M.P., Jurga S., Zaleska-Medynska A. (2017). Highly Visible-Light-Photoactive Heterojunction Based on TiO_2_ Nanotubes Decorated by Pt Nanoparticles and Bi2S3 Quantum Dots. J. Phys. Chem. C.

[18] Mikolajczyk A., Gajewicz A., Mulkiewicz E., Rasulev B., Marchelek M., Diak M., Hirano S., Zaleska-Medynska A., Puzyn T. (2018). Nano-QSAR Modeling for Ecosafe Design of Heterogeneous TiO_2_-Based Nano-Photocatalysts. Environ. Sci. Nano.

[19] Mikolajczyk A., Malankowska A., Nowaczyk G., Gajewicz A., Hirano S., Jurga S., Zaleska-Medynska A., Puzyn T. (2016). Combined Experimental and Computational Approach to Developing Efficient Photocatalysts Based on Au/Pd–TiO_2_ Nanoparticles. Environ. Sci. Nano.

[20] Nevárez-Martínez M.C., Mazierski P., Kobylański M.P., Szczepańska G., Trykowski G., Malankowska A., Kozak M., Espinoza-Montero P.J., Zaleska-Medynska A. (2017). Growth, Structure, and Photocatalytic Properties of Hierarchical V_2_O_5_–TiO_2_ Nanotube Arrays Obtained from the One-Step Anodic Oxidation of Ti–V Alloys. Molecules.

[21] Nischk M., Mazierski P., Gazda M., Zaleska A. (2014). Ordered TiO_2_ Nanotubes: The Effect of Preparation Parameters on the Photocatalytic Activity in Air Purification Process. Appl. Catal. B Environ..

[22] Nischk M., Mazierski P., Wei Z., Siuzdak K., Kouame N.A., Kowalska E., Remita H., Zaleska-Medynska A. (2016). Enhanced Photocatalytic, Electrochemical and Photoelectrochemical Properties of TiO_2_ Nanotubes Arrays Modified with Cu, AgCu and Bi Nanoparticles Obtained via Radiolytic Reduction. Appl. Surf. Sci..

[23] Mazierski P., Lisowski W., Grzyb T., Winiarski M.J., Klimczuk T., Mikołajczyk A., Flisikowski J., Hirsch A., Kołakowska A., Puzyn T. (2017). Enhanced Photocatalytic Properties of Lanthanide-TiO_2_ Nanotubes: An Experimental and Theoretical Study. Appl. Catal. B Environ..

[24] Wang C., Zeng T., Zhu S., Gu C. (2019). Synergistic Mechanism of Rare-Earth Modification TiO_2_ and Photodegradation on Benzohydroxamic Acid. Appl. Sci..

[25] Tobaldi D.M., Pullar R.C., Škapin A.S., Seabra M.P., Labrincha J.A. (2014). Visible Light Activated Photocatalytic Behaviour of Rare Earth Modified Commercial TiO_2_. Mater. Res. Bull..

[26] Nevárez-Martínez M.C., Kobylański M.P., Mazierski P., Wółkiewicz J., Trykowski G., Malankowska A., Kozak M., Espinoza-Montero P.J., Zaleska-Medynska A. (2017). Self-Organized TiO_2_–MnO_2_ Nanotube Arrays for Efficient Photocatalytic Degradation of Toluene. Molecules.

[27] OECD (2014). Guidance Document on the Validation of (Quantitative) Structure-Activity Relationship [(Q)SAR] Models.

[28] Liu Z., Zhang X., Nishimoto S., Murakami T., Fujishima A. (2008). Efficient Photocatalytic Degradation of Gaseous Acetaldehyde by Highly Ordered TiO_2_ Nanotube Arrays. Environ. Sci. Technol..

[29] Lee K., Mazare A., Schmuki P. (2014). One-Dimensional Titanium Dioxide Nanomaterials: Nanotubes. Chem. Rev..

[30] Macak J.M., Zlamal M., Krysa J., Schmuki P. (2007). Self-Organized TiO_2_ Nanotube Layers as Highly Efficient Photocatalysts. Small.

[31] Nakata K., Fujishima A. (2012). TiO_2_ Photocatalysis: Design and Applications. J. Photochem. Photobiol. C Photochem. Rev..

[32] Paramasivam I., Jha H., Liu N., Schmuki P. (2012). A Review of Photocatalysis Using Self-organized TiO_2_ Nanotubes and Other Ordered Oxide Nanostructures. Small.

[33] Paszkiewicz M., Łuczak J., Lisowski W., Patyk P., Zaleska-Medynska A. (2016). The ILs-Assisted Solvothermal Synthesis of TiO_2_ Spheres: The Effect of Ionic Liquids on Morphology and Photoactivity of TiO_2_. Appl. Catal. B Environ..

[34] Lee I., Yang J. (2009). Common Clustering Algorithms. Compr. Chemom..

[35] Python 3. https://www.python.org.

[36] Sosnowska A., Barycki M., Zaborowska M., Rybinska A., Puzyn T. (2014). Towards Designing Environmentally Safe Ionic Liquids: The Influence of the Cation Structure. Green Chem..

[37] Chirico N., Gramatica P. (2011). Real External Predictivity of QSAR Models: How To Evaluate It? Comparison of Different Validation Criteria and Proposal of Using the Concordance Correlation Coefficient. J. Chem. Inf. Model..

[38] Gramatica P., Chirico N., Papa E., Cassani S., Kovarich S. (2013). QSARINS: A New Software for the Development, Analysis, and Validation of QSAR MLR Models. J. Comput. Chem..

