# Supplementary material for: One Step before Synthesis: Structure–Property–Condition Relationship Models to Sustainable Design of Efficient TiO2-Based Multicomponent Nanomaterials
Source: Int J Mol Sci. 2022 Oct 30;23(21):13196. doi: 10.3390/ijms232113196 (PMC9658538; doi:10.3390/ijms232113196)
Supplement: Supplementary file 1 [file ijms-23-13196-s001.zip › ijms-1970453-supplementary.pdf]

Supporting information

One Step before Synthesis: Structure–Property–Condition Relationship Models to Sustainable Design of Efficient TiO_2-Based Multicomponent Nanomaterials

Alicja Mikolajczyk [1,2,*] and Dawid Falkowski [2]

1 Laboratory of Environmental Chemoinformatics, Faculty of Chemistry, University of Gdansk, Wita Stwosza 63, 80-308 Gdansk, Poland
2 QSAR Lab., Trzy Lipy 3, 80-172 Gdansk, Poland
* Correspondence: alicja.mikolajczyk@ug.edu.pl

Table S1. – full virtual data base of modified TiO_2 nanotubes

sample label	Diameter [nm]	Lenght [nm]	Wall thickness [nm]	Ti content in foil [% wt.]	Ag content in foil [% wt.]	V content in foil [% wt.]	Mn content in foil [% wt.]	Au content in foil [% wt.]	Pt content in foil [% wt.]	Pd content in foil [% wt.]	RE content in foil [% wt.]	Number of electrodes	Ultrasound time [min]	Drying temperature [°C]	Anodization voltage [V]	Anodization time [min]	Ethylene glycol volume [%]	H2O volume [%]	IL amount [mol/dm3]	NH4F content [mol/dm3]	RE(NO3)3 content [mol/dm3]	Urea content [% weight]	Calcination time [min]	Calcination temp [°C]	UV-Vis (λ>350 nm) phenol photodegradation rate [μmol·dm-3· min-1]	Vis (λ> 420 nm) phenol photodegradation rate [μmol·dm3· min-1]	Source
IL_[EMIM]	100	350	20	100	0	0	0	0	0	0	0	3	5	80	80	20	0,90	0,10	0,1	0	0	0	60	450	1	0,2	
IL_[BMIM]	110	450	25	100	0	0	0	0	0	0	0	3	5	80	80	20	0,90	0,10	0,1	0	0	0	60	450	1,26	0,3	
IL_[OMIM]	120	700	30	100	0	0	0	0	0	0	0	3	5	80	80	20	0,90	0,10	0,1	0	0	0	60	450	1,53	0,48	
IL_[OMIM]_60V	90	200	15	100	0	0	0	0	0	0	0	3	5	80	60	20	0,90	0,10	0,1	0	0	0	60	450	0,45	0,16	
IL_[OMIM]_70V	110	600	25	100	0	0	0	0	0	0	0	3	5	80	70	20	0,90	0,10	0,1	0	0	0	60	450	1,31	0,41	
IL_[OMIM]_90V	135	850	35	100	0	0	0	0	0	0	0	3	5	80	90	20	0,90	0,10	0,1	0	0	0	60	450	1,82	0,63	
IL_[OMIM]_0,2F-	124	750	32	100	0	0	0	0	0	0	0	3	5	80	80	20	0,90	0,10	0,2	0	0	0	60	450	1,58	0,53	[1]
IL_[OMIM]_0,3F-	129	810	35	100	0	0	0	0	0	0	0	3	5	80	80	20	0,90	0,10	0,3	0	0	0	60	450	1,65	0,55	
IL_[OMIM]_0%H2O	60	200	10	100	0	0	0	0	0	0	0	3	5	80	80	20	1,00	0,00	0,1	0	0	0	60	450	0,63	0,25	
IL_[OMIM]_2,5%H2O	70	300	15	100	0	0	0	0	0	0	0	3	5	80	80	20	0,98	0,03	0,1	0	0	0	60	450	0,76	0,32	
IL_[OMIM]_5,0%H2O	80	650	20	100	0	0	0	0	0	0	0	3	5	80	80	20	0,95	0,05	0,1	0	0	0	60	450	1,4	0,39	
IL_[OMIM]_15%H2O	115	350	25	100	0	0	0	0	0	0	0	3	5	80	80	20	0,85	0,15	0,1	0	0	0	60	450	0,89	0,36	
IL2_pristine_10V				100	0	0	0	0	0	0	0	3	5	80	10	60	0,98	0,02	0	0,2	0	0	60	450	0,99		
IL2_10V	31,8	600	4,6	100	0	0	0	0	0	0	0	3	5	80	10	60	0,98	0,02	0,01	0,2	0	0	60	450	2,3		
IL2_20V	71,1	1030	9,3	100	0	0	0	0	0	0	0	3	5	80	20	60	0,98	0,02	0,01	0,2	0	0	60	450	3,11		
IL2_pristine_30V	91,5	2130	8,4	100	0	0	0	0	0	0	0	3	5	80	30	60	0,98	0,02	0	0,2	0	0	60	450	1,56		
IL2_0.005	101,5	1970	10,2	100	0	0	0	0	0	0	0	3	5	80	30	60	0,98	0,02	0,005	0,2	0	0	60	450	3,48		
IL2_0.007	108,8	2030	12,8	100	0	0	0	0	0	0	0	3	5	80	30	60	0,98	0,02	0,007	0,2	0	0	60	450	3,56		
IL2_0.01	93,1	2050	11,1	100	0	0	0	0	0	0	0	3	5	80	30	60	0,98	0,02	0,01	0,2	0	0	60	450	3,37		[2]
IL2_400	88,5	1990	8,9	100	0	0	0	0	0	0	0	3	5	80	30	60	0,98	0,02	0,01	0,2	0	0	60	400	3,04		
IL2_500	98,7	2050	9,4	100	0	0	0	0	0	0	0	3	5	80	30	60	0,98	0,02	0,01	0,2	0	0	60	500	3,04		
IL2_4%H2O	96,2	1540	12,4	100	0	0	0	0	0	0	0	3	5	80	30	60	0,96	0,04	0,01	0,2	0	0	60	450	3,44		
IL2_6%H2O	98,8	1290	15,1	100	0	0	0	0	0	0	0	3	5	80	30	60	0,94	0,06	0,01	0,2	0	0	60	450	3,49		
IL2_10%H2O	103,9	1220	16,1	100	0	0	0	0	0	0	0	3	5	80	30	60	0,90	0,10	0,01	0,2	0	0	60	450	3,54		
IL2_15%H2O	114,5	1050	13,1	100	0	0	0	0	0	0	0	3	5	80	30	60	0,85	0,15	0,01	0,2	0	0	60	450	3,58		
MM_NT				100	0	0	0	0	0	0	0	2	5	80	30	60	0,98	0,02	0,00	0,09	0	0	60	450			
MM_Ag-NT	90	2300	16	100	0	0	0	0	0	0	0	2	5	80	30	60	0,98	0,02	0,00	0,09	0	0	60	450			
MM_Cu-NT_I	90	2300	16	100	0	0	0	0	0	0	0	2	5	80	30	60	0,98	0,02	0,00	0,09	0	0	60	450			
MM_Cu-NT_II	90	2300	16	100	0	0	0	0	0	0	0	2	5	80	30	60	0,98	0,02	0,00	0,09	0	0	60	450			
MM_Cu-NT_III	90	2300	16	100	0	0	0	0	0	0	0	2	5	80	30	60	0,98	0,02	0,00	0,09	0	0	60	450			[3]
MM_Cu-NT_IV	90	2300	16	100	0	0	0	0	0	0	0	2	5	80	30	60	0,98	0,02	0,00	0,09	0	0	60	450			
MM_AgCu-NT_I	90	2300	16	100	0	0	0	0	0	0	0	2	5	80	30	60	0,98	0,02	0,00	0,09	0	0	60	450			
MM_AgCu-NT_II	90	2300	16	100	0	0	0	0	0	0	0	2	5	80	30	60	0,98	0,02	0,00	0,09	0	0	60	450			
MM_AgCu-NT_III	90	2300	16	100	0	0	0	0	0	0	0	2	5	80	30	60	0,98	0,02	0,00	0,09	0	0	60	450			

sample label	Diameter [nm]	Lenght [nm]	Wall thickness [nm]	Ti content in foil [% wt.]	Ag content in foil [% wt.]	V content in foil [% wt.]	Mn content in foil [% wt.]	Au content in foil [% wt.]	Pt content in foil [% wt.]	Pd content in foil [% wt.]	RE content in foil [% wt.]	Number of electrodes	Ultrasound time [min]	Drying temperature [°C]	Anodization voltage [V]	Anodization time [min]	Ethylene glycol volume [%]	H2O volume [%]	IL amount [mol/dm3]	NH4F content [mol/dm3]	RE(NO3)3 content [mol/dm3]	Urea content [% weight]	Calcination time [min]	Calcination temp [°C]	UV-Vis (λ>350 nm) phenol photodegradation rate [μmol·dm-3· min-1]	Vis (λ> 420 nm) phenol photodegradation rate [μmol·dm3· min-1]	Source
MM_AgCu-NT_IV	90	2300	16	100	0	0	0	0	0	0	0	2	5	80	30	60	0,98	0,02	0,00	0,09	0	0	60	450			
MM_Bi-NT_I	90	2300	16	100	0	0	0	0	0	0	0	2	5	80	30	60	0,98	0,02	0,00	0,09	0	0	60	450			
MM_Bi-NT_II	90	2300	16	100	0	0	0	0	0	0	0	2	5	80	30	60	0,98	0,02	0,00	0,09	0	0	60	450			
MM_Bi-NT_III	90	2300	16	100	0	0	0	0	0	0	0	2	5	80	30	60	0,98	0,02	0,00	0,09	0	0	60	450			
MM_Bi-NT_IV	90	2300	16	100	0	0	0	0	0	0	0	2	5	80	30	60	0,98	0,02	0,00	0,09	0	0	60	450			
NT_20V_1h	54	800	6	100	0	0	0	0	0	0	0	2	5	80	20	60	0,98	0,02	0,00	0,09	0	0	60	450	1,86	0,05	
NT_30V_1h	65	1700	6	100	0	0	0	0	0	0	0	2	5	80	30	60	0,98	0,02	0,00	0,09	0	0	60	450	2,02	0,16	
NT_40V_1h	72	2200	6	100	0	0	0	0	0	0	0	2	5	80	40	60	0,98	0,02	0,00	0,09	0	0	60	450	4,25	0,37	
NT_50V_1h	78	2700	14	100	0	0	0	0	0	0	0	2	5	80	50	60	0,98	0,02	0,00	0,09	0	0	60	450	2,13	0,27	
NT_40V_0.5h	66	1600	6	100	0	0	0	0	0	0	0	2	5	80	40	30	0,98	0,02	0,00	0,09	0	0	60	450	2,1	0,11	
NT_40V_2h	96	3800	11,5	100	0	0	0	0	0	0	0	2	5	80	40	120	0,98	0,02	0,00	0,09	0	0	60	450	2,23	0,12	
N-NT_0.2%_20V_1h	61	1000	10,5	100	0	0	0	0	0	0	0	2	5	80	20	60	0,98	0,02	0,00	0,09	0	0,2	60	450	2,13	0,27	
N-NT_0.2%_30V_1h	76	2000	11	100	0	0	0	0	0	0	0	2	5	80	30	60	0,98	0,02	0,00	0,09	0	0,2	60	450	2,34	0,69	[4]
N-NT_0.2%_40V_1h	89	3500	10,5	100	0	0	0	0	0	0	0	2	5	80	40	60	0,98	0,02	0,00	0,09	0	0,2	60	450	4,53	1,33	
N-NT_0.2%_50V_1h	114	6800	9,5	100	0	0	0	0	0	0	0	2	5	80	50	60	0,98	0,02	0,00	0,09	0	0,2	60	450	3,45	1,01	
N-NT_0.2%_40V_0.5h	70	1600	7	100	0	0	0	0	0	0	0	2	5	80	40	30	0,98	0,02	0,00	0,09	0	0,2	60	450	2,39	0,53	
N-NT_0.2%_40V_2h	112	4800	13,5	100	0	0	0	0	0	0	0	2	5	80	40	120	0,98	0,02	0,00	0,09	0	0,2	60	450	3,72	1,06	
N-NT_0.1%_40V_1h	72	3500	4,5	100	0	0	0	0	0	0	0	2	5	80	40	60	0,98	0,02	0,00	0,09	0	0,1	60	450	4,25	0,96	
N-NT_0.3%_40V_1h	63	3400	4,5	100	0	0	0	0	0	0	0	2	5	80	40	60	0,98	0,02	0,00	0,09	0	0,3	60	450	4,36	0,8	
N-NT_0.5%_40V_1h	71	3400	6	100	0	0	0	0	0	0	0	2	5	80	40	60	0,98	0,02	0,00	0,09	0	0,5	60	450	4,09	0,48	
REE_base	85	2500	15	100	0	0	0	0	0	0	0	2	5	80	30	60	0,98	0,02	0	0,1	0,025	0	60	450			
REE_Er	85	2500	15	100	0	0	0	0	0	0	0	2	5	80	30	60	0,98	0,02	0	0,1	0,025	0	60	450			
REE_Yb	85	2500	15	100	0	0	0	0	0	0	0	2	5	80	30	60	0,98	0,02	0	0,1	0,025	0	60	450			
REE_Ho	85	2500	15	100	0	0	0	0	0	0	0	2	5	80	30	60	0,98	0,02	0	0,1	0,025	0	60	450			[5]
REE_Tb	85	2500	15	100	0	0	0	0	0	0	0	2	5	80	30	60	0,98	0,02	0	0,1	0,025	0	60	450			
REE_Gd	85	2500	15	100	0	0	0	0	0	0	0	2	5	80	30	60	0,98	0,02	0	0,1	0,025	0	60	450			
REE_Pr	85	2500	15	100	0	0	0	0	0	0	0	2	5	80	30	60	0,98	0,02	0	0,1	0,025	0	60	450			
REE2_Ti_30V	80	1500	10	100	0	0	0	0	0	0	0	2	5	80	40	60	0,98	0,02	0	0,09		0	60	450	1,25	0,04	
REE2_Ti_40V	100	3000	13	100	0	0	0	0	0	0	0	2	5	80	40	60	0,98	0,02	0	0,09		0	60	450	1,35	0,13	
REE2_Ti_50V	120	6000	18	100	0	0	0	0	0	0	0	2	5	80	40	60	0,98	0,02	0	0,09		0	60	450	1,44	0,15	
REE2_Ti90Ho10_40V	99	5600	19	90	0	0	0	0	0	0	10	2	5	80	40	60	0,98	0,02	0	0,09		0	60	450	2,37	0,45	
REE2_Ti90Er10_40V	98	5500	19	90	0	0	0	0	0	0	10	2	5	80	40	60	0,98	0,02	0	0,09		0	60	450	1,82	0,43	[6]
REE2_Ti90Nd10_40V	98	5300	18	90	0	0	0	0	0	0	10	2	5	80	40	60	0,98	0,02	0	0,09		0	60	450	3,04	0,4	
REE2_Ti90Y10_40V	98	4500	18	90	0	0	0	0	0	0	10	2	5	80	40	60	0,98	0,02	0	0,09		0	60	450	2,12	0,13	
REE2_Ti90Ce10_40V	96	4600	17	90	0	0	0	0	0	0	10	2	5	80	40	60	0,98	0,02	0	0,09		0	60	450	3,09	0	
REE2_Ti90Tm10_40V	95	4800	17	90	0	0	0	0	0	0	10	2	5	80	40	60	0,98	0,02	0	0,09		0	60	450	2,96	0,26	

sample label	Diameter [nm]	Lenght [nm]	Wall thickness [nm]	Ti content in foil [% wt.]	Ag content in foil [% wt.]	V content in foil [% wt.]	Mn content in foil [% wt.]	Au content in foil [% wt.]	Pt content in foil [% wt.]	Pd content in foil [% wt.]	RE content in foil [% wt.]	Number of electrodes	Ultrasound time [min]	Drying temperature [°C]	Anodization voltage [V]	Anodization time [min]	Ethylene glycol volume [%]	H2O volume [%]	IL amount [mol/dm3]	NH4F content [mol/dm3]	RE(NO3)3 content [mol/dm3]	Urea content [% weight]	Calcination time [min]	Calcination temp [°C]	UV-Vis (λ>350 nm) phenol photodegradation rate [µmol·dm-3· min-1]	Vis (λ> 420 nm) phenol photodegradation rate [µmol·dm3· min-1]	Source
Ag2O_Ti_30V	80	1500	10	100	0	0	0	0	0	0	0	3	5	80	30	60	0,98	0,02	0	0,09		0	60	450	1,25	0,04	
Ag2O_Ti_40V	100	3000	13	100	0	0	0	0	0	0	0	3	5	80	40	60	0,98	0,02	0	0,09		0	60	450	1,35	0,13	
Ag2O_Ti_50V	120	6000	18	100	0	0	0	0	0	0	0	3	5	80	50	60	0,98	0,02	0	0,09		0	60	450	1,44	0,15	
Ag2O_Ti95Ag5_30V	70	1500	12	95	5	0	0	0	0	0	0	3	5	80	30	60	0,98	0,02	0	0,09		0	60	450	1,61	0,30	
Ag2O_Ti90Ag10_30V	70	1500	12	90	10	0	0	0	0	0	0	3	5	80	30	60	0,98	0,02	0	0,09		0	60	450	2,52	0,38	[7]
Ag2O_Ti85Ag15_30V	70	1500	12	85	15	0	0	0	0	0	0	3	5	80	30	60	0,98	0,02	0	0,09		0	60	450	1,83	0,50	
Ag2O_Ti90Ag10_40V	90	3000	15	90	10	0	0	0	0	0	0	3	5	80	40	60	0,98	0,02	0	0,09		0	60	450	2,66	0,50	
Ag2O_Ti90Ag10_50V	115	6000	20	90	10	0	0	0	0	0	0	3	5	80	50	60	0,98	0,02	0	0,09		0	60	450	2,89	0,41	
BiPt_NTs*	85	2500	15	100	0	0	0	0	0	0	0	3	5	80	40	60	0,98	0,02	0	0,09		0	60	450			
BiPt_1Pt-NTs	85	2500	15	100	0	0	0	0	0	0	0	3	5	80	40	60	0,98	0,02	0	0,09		0	60	450			
BiPt_Pt-NTs*	85	2500	15	100	0	0	0	0	0	0	0	3	5	80	40	60	0,98	0,02	0	0,09		0	60	450			
BiPt_2Pt-NTs	85	2500	15	100	0	0	0	0	0	0	0	3	5	80	40	60	0,98	0,02	0	0,09		0	60	450			[8]
BiPt_2Bi2S3-NTs	85	2500	15	100	0	0	0	0	0	0	0	3	5	80	40	60	0,98	0,02	0	0,09		0	60	450			
BiPt_Bi2S3-NTs*	85	2500	15	100	0	0	0	0	0	0	0	3	5	80	40	60	0,98	0,02	0	0,09		0	60	450			
BiPt_6Bi2S3-NTs	85	2500	15	100	0	0	0	0	0	0	0	3	5	80	40	60	0,98	0,02	0	0,09		0	60	450			
BiPt_Bi2S3-Pt-NTs*	85	2500	15	100	0	0	0	0	0	0	0	3	5	80	40	60	0,98	0,02	0	0,09		0	60	450			
IL4_0.05	107	6000	5,4	100	0	0	0	0	0	0	0	3	5	80	40	60	0,97	0,03	0,005	0,2		0	60	450	5,53		
IL4_0.1	114	6200	9,4	100	0	0	0	0	0	0	0	3	5	80	40	60	0,96	0,04	0,01	0,2		0	60	450	9,12		
IL4_0.2	115	6200	10	100	0	0	0	0	0	0	0	3	5	80	40	60	0,95	0,05	0,021	0,2		0	60	450	8,35		[9]
IL4_0.3	119	6800	11	100	0	0	0	0	0	0	0	3	5	80	40	60	0,94	0,06	0,031	0,2		0	60	450	8,45		
IL4_0.5	133	7200	13	100	0	0	0	0	0	0	0	3	5	80	40	60	0,93	0,07	0,051	0,2		0	60	450	8,38		
IL4_1.0	140	8100	15	100	0	0	0	0	0	0	0	3	5	80	40	60	0,92	0,08	0,103	0,2		0	60	450	8,59		
MnO_Ti_30V	81	1500	10	100	0	0	0	0	0	0	0	3	1	80	30	60	0,98	0,02	0	0,09		0	60	450	7,1		
MnO_Ti_40V	100	5000	13	100	0	0	0	0	0	0	0	3	1	80	40	60	0,98	0,02	0	0,09		0	60	450			
MnO_Ti_50V	120	16200	18	100	0	0	0	0	0	0	0	3	1	80	50	60	0,98	0,02	0	0,09		0	60	450			
MnO_Ti90Mn10_30V	76	1000	8	90	0	0	10	0	0	0	0	3	1	80	30	60	0,98	0,02	0	0,09		0	60	450			
MnO_Ti90Mn10_40V	92	1500	9	90	0	0	10	0	0	0	0	3	1	80	40	60	0,98	0,02	0	0,09		0	60	450			[10]
MnO_Ti90Mn10_50V	118	2800	9	90	0	0	10	0	0	0	0	3	1	80	50	60	0,98	0,02	0	0,09		0	60	450			
MnO_Ti85Mn15_40V_2%	94	1300	9	85	0	0	15	0	0	0	0	3	1	80	40	60	0,98	0,02	0	0,09		0	60	450			
MnO_Ti85Mn15_40V_5%	90	1300	9	85	0	0	15	0	0	0	0	3	1	80	40	60	0,95	0,05	0	0,09		0	60	450			
MnO_Ti85Mn15_40V_10%	115	1100	11	85	0	0	15	0	0	0	0	3	1	80	40	60	0,9	0,1	0	0,09		0	60	450			
MnO_Ti95Mn5_40V	94	3400	9	95	0	0	5	0	0	0	0	3	1	80	40	60	0,98	0,02	0	0,09		0	60	450			
V2O5_Ti90V10_30V	61	800	13	90	0	10	0	0	0	0	0	3	1	80	30	60	0,98	0,02	0	0,09		0	60	450			
V2O5_Ti90V10_40V	91	300	19	90	0	10	0	0	0	0	0	3	1	80	40	60	0,98	0,02	0	0,09		0	60	450			[11]
V2O5_Ti90V10_50V	101	400	30	90	0	10	0	0	0	0	0	3	1	80	50	60	0,98	0,02	0	0,09		0	60	450			
V2O5_Ti85V15_40V_2%				85	0	15	0	0	0	0	0	3	1	80	40	60	0,98	0,02	0	0,09		0	60	450			

sample label	Diameter [nm]	Lenght [nm]	Wall thickness [nm]	Ti content in foil [% wt.]	Ag content in foil [% wt.]	V content in foil [% wt.]	Mn content in foil [% wt.]	Au content in foil [% wt.]	Pt content in foil [% wt.]	Pd content in foil [% wt.]	RE content in foil [% wt.]	Number of electrodes	Ultrasound time [min]	Drying temperature [°C]	Anodization voltage [V]	Anodization time [min]	Ethylene glycol volume [%]	H2O volume [%]	IL amount [mol/dm3]	NH4F content [mol/dm3]	RE(NO3)3 content [mol/dm3]	Urea content [% weight]	Calcination time [min]	Calcination temp [°C]	UV-Vis (λ>350 nm) phenol photodegradation rate [µmol·dm-3· min-1]	Vis (λ> 420 nm) phenol photodegradation rate [µmol·dm3· min-1]	Source
V2O5_Ti85V15_40V_5%	103	900	20	85	0	15	0	0	0	0	0	3	1	80	40	60	0,95	0,05	0	0,09		0	60	450			
V2O5_Ti85V15_40V_10%				85	0	15	0	0	0	0	0	3	1	80	40	60	0,9	0,1	0	0,09		0	60	450			
V2O5_Ti95V5_40V	86	1000	11	95	0	5	0	0	0	0	0	3	1	80	40	60	0,98	0,02	0	0,09		0	60	450			
V2O5_Ti_30V	81	1500	10	100	0	0	0	0	0	0	0	3	1	80	30	60	0,98	0,02	0	0,09		0	60	450			
V2O5_Ti_40V	100	5000	13	100	0	0	0	0	0	0	0	3	1	80	40	60	0,98	0,02	0	0,09		0	60	450			
V2O5_Ti_50V	120	16200	18	100	0	0	0	0	0	0	0	3	1	80	50	60	0,98	0,02	0	0,09		0	60	450			

Table S2. – The database of experimental condition and photocatalytic activity under UV-Vis light of investigated TiO_2-based NTs

sample label	Diameter [nm]	Lenght [nm]	Wall thickness [nm]	Ti content in foil [% wt.]	Ag content in foil [% wt.]	RE content in foil [% wt.]	Foil surface [cm^2]	Number of electrodes	Anodization voltage [V]	Anodization time [min]	Ethylene glycol volume [%]	H2O volume [%]	IL amount [mol/dm^3]	NH4F content [mol/dm^3]	Urea content [% weight]	Calcination temp [°C]	UV-Vis (λ>350 nm) phenol photodegradation rate [μmol·dm-3· min-1]	Vis (λ> 420 nm) phenol photodegradation rate [μmol·dm3· min-1]
IL_[EMIM]	100	350	20	100	0	0	6	3	80	20	0,9	0,1	0,1	0	0	450	1	0,2
IL_[BMIM]	110	450	25	100	0	0	6	3	80	20	0,9	0,1	0,1	0	0	450	1,26	0,3
IL_[OMIM]	120	700	30	100	0	0	6	3	80	20	0,9	0,1	0,1	0	0	450	1,53	0,48
IL_[OMIM]_60V	90	200	15	100	0	0	6	3	60	20	0,9	0,1	0,1	0	0	450	0,45	0,16
IL_[OMIM]_70V	110	600	25	100	0	0	6	3	70	20	0,9	0,1	0,1	0	0	450	1,31	0,41
IL_[OMIM]_90V	135	850	35	100	0	0	6	3	90	20	0,9	0,1	0,1	0	0	450	1,82	0,63
IL_[OMIM]_0,2F-	124	750	32	100	0	0	6	3	80	20	0,9	0,1	0,2	0	0	450	1,58	0,53
IL_[OMIM]_0,3F-	129	810	35	100	0	0	6	3	80	20	0,9	0,1	0,3	0	0	450	1,65	0,55
IL_[OMIM]_0%H2O	60	200	10	100	0	0	6	3	80	20	1	0	0,1	0	0	450	0,63	0,25
IL_[OMIM]_2,5%H2O	70	300	15	100	0	0	6	3	80	20	0,98	0,03	0,1	0	0	450	0,76	0,32
IL_[OMIM]_5,0%H2O	80	650	20	100	0	0	6	3	80	20	0,95	0,05	0,1	0	0	450	1,4	0,39
IL_[OMIM]_15%H2O	115	350	25	100	0	0	6	3	80	20	0,85	0,15	0,1	0	0	450	0,89	0,36
IL2_10V	31,8	600	4,6	100	0	0	6	3	10	60	0,98	0,02	0,01	0,2	0	450	2,3	
IL2_20V	71,1	1030	9,3	100	0	0	6	3	20	60	0,98	0,02	0,01	0,2	0	450	3,11	
IL2_0.005	101,5	1970	10,2	100	0	0	6	3	30	60	0,98	0,02	0,005	0,2	0	450	3,48	
IL2_0.007	108,8	2030	12,8	100	0	0	6	3	30	60	0,98	0,02	0,007	0,2	0	450	3,56	
IL2_0.01	93,1	2050	11,1	100	0	0	6	3	30	60	0,98	0,02	0,01	0,2	0	450	3,37	
IL2_400	88,5	1990	8,9	100	0	0	6	3	30	60	0,98	0,02	0,01	0,2	0	400	3,04	
IL2_500	98,7	2050	9,4	100	0	0	6	3	30	60	0,98	0,02	0,01	0,2	0	500	3,04	
IL2_4%H2O	96,2	1540	12,4	100	0	0	6	3	30	60	0,96	0,04	0,01	0,2	0	450	3,44	
IL2_6%H2O	98,8	1290	15,1	100	0	0	6	3	30	60	0,94	0,06	0,01	0,2	0	450	3,49	
IL2_10%H2O	103,9	1220	16,1	100	0	0	6	3	30	60	0,9	0,1	0,01	0,2	0	450	3,54	
IL2_15%H2O	114,5	1050	13,1	100	0	0	6	3	30	60	0,85	0,15	0,01	0,2	0	450	3,58	
N-NT_0.2%_20V_1h	61	1000	10,5	100	0	0	6	2	20	60	0,98	0,02	0	0,09	0,2	450	2,13	0,27
N-NT_0.2%_30V_1h	76	2000	11	100	0	0	6	2	30	60	0,98	0,02	0	0,09	0,2	450	2,34	0,69
N-NT_0.2%_40V_1h	89	3500	10,5	100	0	0	6	2	40	60	0,98	0,02	0	0,09	0,2	450	4,53	1,33
N-NT_0.2%_50V_1h	114	6800	9,5	100	0	0	6	2	50	60	0,98	0,02	0	0,09	0,2	450	3,45	1,01
N-NT_0.2%_40V_0.5h	70	1600	7	100	0	0	6	2	40	30	0,98	0,02	0	0,09	0,2	450	2,39	0,53
N-NT_0.2%_40V_2h	112	4800	13,5	100	0	0	6	2	40	120	0,98	0,02	0	0,09	0,2	450	3,72	1,06
N-NT_0.1%_40V_1h	72	3500	4,5	100	0	0	6	2	40	60	0,98	0,02	0	0,09	0,1	450	4,25	0,96
N-NT_0.3%_40V_1h	63	3400	4,5	100	0	0	6	2	40	60	0,98	0,02	0	0,09	0,3	450	4,36	0,8
N-NT_0.5%_40V_1h	71	3400	6	100	0	0	6	2	40	60	0,98	0,02	0	0,09	0,5	450	4,09	0,48
REE2_Ti90Ho10_40V	99	5600	19	90	0	10	6,25	2	40	60	0,98	0,02	0	0,09	0	450	2,37	0,45

sample label	Diameter [nm]	Lenght [nm]	Wall thickness [nm]	Ti content in foil [% wt.]	Ag content in foil [% wt.]	RE content in foil [% wt.]	Foil surface [cm^2]	Number of electrodes	Anodization voltage [V]	Anodization time [min]	Ethylene glycol volume [%]	H_2O volume [%]	IL amount [mol/dm^3]	NH_4F content [mol/dm^3]	Urea content [% weight]	Calcination temp [°C]	UV-Vis (λ>350 nm) phenol photodegradation rate [μmol·dm-3· min-1]	Vis (λ> 420 nm) phenol photodegradation rate [μmol·dm3· min-1]
REE2_Ti90Er10_40V	98	5500	19	90	0	10	6,25	2	40	60	0,98	0,02	0	0,09	0	450	1,82	0,43
REE2_Ti90Nd10_40V	98	5300	18	90	0	10	6,25	2	40	60	0,98	0,02	0	0,09	0	450	3,04	0,4
REE2_Ti90Y10_40V	98	4500	18	90	0	10	6,25	2	40	60	0,98	0,02	0	0,09	0	450	2,12	0,13
REE2_Ti90Ce10_40V	96	4600	17	90	0	10	6,25	2	40	60	0,98	0,02	0	0,09	0	450	3,09	0
REE2_Ti90Tm10_40V	95	4800	17	90	0	10	6,25	2	40	60	0,98	0,02	0	0,09	0	450	2,96	0,26
Ag2O_Ti95Ag5_30V	70	1500	12	95	5	0	6	3	30	60	0,98	0,02	0	0,09	0	450	1,61	0,3
Ag2O_Ti90Ag10_30V	70	1500	12	90	10	0	6	3	30	60	0,98	0,02	0	0,09	0	450	2,52	0,38
Ag2O_Ti85Ag15_30V	70	1500	12	85	15	0	6	3	30	60	0,98	0,02	0	0,09	0	450	1,83	0,5
Ag2O_Ti90Ag10_40V	90	3000	15	90	10	0	6	3	40	60	0,98	0,02	0	0,09	0	450	2,66	0,5
Ag2O_Ti90Ag10_50V	115	6000	20	90	10	0	6	3	50	60	0,98	0,02	0	0,09	0	450	2,89	0,41
IL4_0.05	107	6000	5,4	100	0	0	6	3	40	60	0,97	0,03	0,005	0,2	0	450	9,12	
IL4_0.1	114	6200	9,4	100	0	0	6	3	40	60	0,96	0,04	0,01	0,2	0	450	8,35	
IL4_0.2	115	6200	10	100	0	0	6	3	40	60	0,95	0,05	0,021	0,2	0	450	8,45	
IL4_0.3	119	6800	11	100	0	0	6	3	40	60	0,94	0,06	0,031	0,2	0	450	8,38	
IL4_0.5	133	7200	13	100	0	0	6	3	40	60	0,93	0,07	0,051	0,2	0	450	8,59	
IL4_1.0	140	8100	15	100	0	0	6	3	40	60	0,92	0,08	0,103	0,2	0	450	7,1	

$\boldsymbol{y_i}$ − observed dependent variable
$\boldsymbol{\hat{y}_i}$ − calculated dependent variable
$\boldsymbol{\bar{y}}$ − mean value of the dependent variable
$\boldsymbol{\hat{y}_{i/i}}$ − response of the i-th object estimated by using a model obtained without using the i-th object
$\boldsymbol{\bar{\hat{y}}}$ − average of all $\hat{y}_i$
$\boldsymbol{\bar{\hat{y}}_{CV}}$ − is the average of all $\hat{y}_{i/i}$
$\boldsymbol{\bar{y}_{TR}}$ − average of training observed responses
$\boldsymbol{\bar{y}_{EXT}}$ − average of external observed responses
$\boldsymbol{n_{EXT}}$ − number of external samples
$\boldsymbol{n_{TR}}$ − number of training samples
$\boldsymbol{p}$ − number of predictor variables

Table S3 – list of equations for calculated model statistics

Statistic	Source	Definition	Equation
**R^2**	[12]	Coefficient of determination	$R^2 = 1 - \frac{\sum_{i=1}^{n_{EXT}}(y_i - \hat{y}_i)^2}{\sum_{i=1}^{n_{EXT}}(y_i - \bar{y})^2}$
**$RMSE_C$ (RMSEtr)**	[13]	Root Mean Square Error in fitting	$RMSE_C = \sqrt{\frac{\sum(y_i - \hat{y}_i)^2}{n_{EXT}}}$
**R_{adj}^2**	[13]	Adjusted R^2	$R_{adj}^2 = (1 - R^2) * \left(\frac{n-1}{n-p}\right)$
**CCC_{tr}**	[12]	Concordance correlation coefficient calculated over the training set	$CCC_{tr} = \frac{2\sum_{i=1}^{n_{EXT}}(y_i - \bar{y})(\hat{y}_i - \bar{\hat{y}})}{\sum_{i=1}^{n_{EXT}}(y_i - \bar{y})^2 + \sum_{i=1}^{n_{EXT}}(\hat{y}_i - \bar{\hat{y}})^2 + n_{EXT}(\bar{y} - \bar{\hat{y}})^2}$
**MAE_{tr} (MAE calc)**	[12]	Mean Absolute Error using on fitting	$MAE_{tr} = \frac{\sum\lvert y_i - \hat{y}_i\rvert}{n_{EXT}}$
**Q^2_{LOO}**	[12]	Correlation coefficient for leave-one-out cross-validation	$Q_{LOO}^2 = 1 - \frac{\sum_{i=1}^{n_{EXT}}(y_i - \hat{y}_{i/i})^2}{\sum_{i=1}^{n_{EXT}}(y_i - \bar{y})^2}$
**$RMSE_{CV}$**	[13]	Root Mean Square Error using cross validation prediction	$RMSE_{CV} = \sqrt{\frac{\sum(y_i - \hat{y}_{i/i})^2}{n_{EXT}}}$
**CCC_{CV}**	[13]	Concordance correlation coefficient calculated in cross validation	$CCC_{CV} = \frac{2\sum_{i=1}^{n_{EXT}}(y_i - \bar{y})(\hat{y}_{i/i} - \bar{\hat{y}}_{CV})}{\sum_{i=1}^{n_{EXT}}(y_i - \bar{y})^2 + \sum_{i=1}^{n_{EXT}}(\hat{y}_{i/i} - \bar{\hat{y}}_{CV})^2 + n_{EXT}(\bar{y} - \bar{\hat{y}}_{CV})^2}$
**MAE_{CV}**	[13]	Mean Absolute Error using cross validation predictions	$MAE_{CV} = \frac{\sum\lvert y_i - \hat{y}_{i/i}\rvert}{n_{EXT}}$
**Q^2_{F1}**	[12]	External validation coefficient	$Q_{F1}^2 = 1 - \frac{\sum_{i=1}^{n_{EXT}}(\hat{y}_i - y_i)^2}{\sum_{i=1}^{n_{EXT}}(y_i - \bar{y}_{TR})^2}$
**Q^2_{F2}**	[12]	External validation coefficient	$Q_{F2}^2 = 1 - \frac{\sum_{i=1}^{n_{EXT}}(\hat{y}_i - y_i)^2}{\sum_{i=1}^{n_{EXT}}(y_i - \bar{y}_{EXT})^2}$

Statistic	Source	Definition	Equation
**Q^2_{F3}**	[12]	External validation coefficient	$Q^2_{F3} = 1 - \frac{\frac{\sum_{i=1}^{n_{EXT}}(\hat{y}_i - y_i)^2}{n_{EXT}}}{\frac{\sum_{i=1}^{n_{EXT}}(y_i - \bar{y}_{TR})^2}{n_{TR}}}$
**R_{EXT}^2**	[13]	External determination coefficient	$R^2_{EXT} = 1 - \frac{\sum_{i=1}^{n_{EXT}}(\hat{y}_i - y_i)^2}{\sum_{i=1}^{n_{EXT}}(y_i - \bar{y})^2}$
**$RMSE_{EXT}$ (RMSEP)**	[13]	Root Mean Square Error in external prediction	$RMSE_{EXT} = \sqrt{\frac{\sum_{i=1}^{n_{EXT}}(\hat{y}_i - y_i)^2}{n_{EXT}}}$
**CCC_{EXT}**	[13]	Concordance correlation coefficient	$CCC_{EXT} = \frac{2\sum_{i=1}^{n_{EXT}}(y_i - \bar{y})(\hat{y}_i - \bar{\hat{y}})}{\sum_{i=1}^{n_{EXT}}(y_i - \bar{y})^2 + \sum_{i=1}^{n_{EXT}}(\hat{y}_i - \bar{\hat{y}})^2 + n_{EXT}(\bar{y} - \bar{\hat{y}})^2}$
**MAE_{EXT}**	[12]	Mean Absolute Error in external prediction	$MAE_{EXT} = \frac{\sum_{i=1}^{n_{EXT}}\lvert\hat{y}_i - y_i\rvert}{n_{EXT}}$
**F**	[13]	F-value	$F = \frac{\frac{\sum_{i=1}^{n_{EXT}}(\hat{y}_i - \bar{y})^2}{p}}{\frac{(\hat{y}_i - y_i)^2}{n - p - 1}}$

1. Mazierski, P.; Łuczak, J.; Lisowski, W.; Winiarski, M.J.; Klimczuk, T.; Zaleska-Medynska, A. The ILs-Assisted Electrochemical Synthesis of TiO2 Nanotubes: The Effect of Ionic Liquids on Morphology and Photoactivity. *Appl Catal B Environ* **2017**, *214*, 100–113, doi:10.1016/j.apcatb.2017.05.005.

2. Pancielejko, A.; Mazierski, P.; Lisowski, W.; Zaleska-Medynska, A.; Łuczak, J. Ordered TiO2 Nanotubes with Improved Photoactivity through Self-Organizing Anodization with the Addition of an Ionic Liquid: Effects of the Preparation Conditions. *Acs Sustain Chem Eng* **2019**, *7*, 15585–15596, doi:10.1021/acssuschemeng.9b03589.

3. Nischk, M.; Mazierski, P.; Wei, Z.; Siuzdak, K.; Kouame, N.A.; Kowalska, E.; Remita, H.; Zaleska-Medynska, A. Enhanced Photocatalytic, Electrochemical and Photoelectrochemical Properties of TiO2 Nanotubes Arrays Modified with Cu, AgCu and Bi Nanoparticles Obtained via Radiolytic Reduction. *Appl Surf Sci* **2016**, *387*, 89–102, doi:10.1016/j.apsusc.2016.06.066.

4. Mazierski, P.; Nischk, M.; Gołkowska, M.; Lisowski, W.; Gazda, M.; Winiarski, M.J.; Klimczuk, T.; Zaleska-Medynska, A. Photocatalytic Activity of Nitrogen Doped TiO2 Nanotubes Prepared by Anodic Oxidation: The Effect of Applied Voltage, Anodization Time and Amount of Nitrogen Dopant. *Appl Catal B Environ* **2016**, *196*, 77–88, doi:10.1016/j.apcatb.2016.05.006.

5. Mazierski, P.; Lisowski, W.; Grzyb, T.; Winiarski, M.J.; Klimczuk, T.; Mikołajczyk, A.; Flisikowski, J.; Hirsch, A.; Kołakowska, A.; Puzyn, T.; et al. Enhanced Photocatalytic Properties of Lanthanide-TiO2 Nanotubes: An Experimental and Theoretical Study. *Appl Catal B Environ* **2017**, *205*, 376–385, doi:10.1016/j.apcatb.2016.12.044.

6. Parnicka, P.; Mazierski, P.; Lisowski, W.; Klimczuk, T.; Nadolna, J.; Zaleska-Medynska, A. A New Simple Approach to Prepare Rare-Earth Metals-Modified TiO2 Nanotube Arrays Photoactive under Visible Light: Surface Properties and Mechanism Investigation. *Results Phys* **2019**, *12*, 412–423, doi:10.1016/j.rinp.2018.11.073.

7. Mazierski, P.; Malankowska, A.; Kobylański, M.; Diak, M.; Kozak, M.; Winiarski, M.J.; Klimczuk, T.; Lisowski, W.; Nowaczyk, G.; Zaleska-Medynska, A. Photocatalytically Active TiO2/Ag2O Nanotube Arrays Interlaced with Silver Nanoparticles Obtained from the One-Step Anodic Oxidation of Ti–Ag Alloys. *Acs Catal* **2017**, *7*, 2753–2764, doi:10.1021/acscatal.7b00056.

8. Mazierski, P.; Nadolna, J.; Nowaczyk, G.; Lisowski, W.; Winiarski, M.J.; Klimczuk, T.; Kobylański, M.P.; Jurga, S.; Zaleska-Medynska, A. Highly Visible-Light-Photoactive Heterojunction Based on TiO2 Nanotubes Decorated by Pt Nanoparticles and Bi2S3 Quantum Dots. *J Phys Chem C* **2017**, *121*, 17215–17225, doi:10.1021/acs.jpcc.7b03895.

9. Pancielejko, A.; Mazierski, P.; Lisowski, W.; Zaleska-Medynska, A.; Kosek, K.; Łuczak, J. Facile Formation of Self-Organized TiO2 Nanotubes in Electrolyte Containing Ionic Liquid-Ethylammonium Nitrate and Their Remarkable Photocatalytic Properties. *Acs Sustain Chem Eng* **2018**, *6*, 14510–14522, doi:10.1021/acssuschemeng.8b03154.

10. Nevárez-Martínez, M.C.; Kobylański, M.P.; Mazierski, P.; Wółkiewicz, J.; Trykowski, G.; Malankowska, A.; Kozak, M.; Espinoza-Montero, P.J.; Zaleska-Medynska, A. Self-Organized TiO2–MnO2 Nanotube Arrays for Efficient Photocatalytic Degradation of Toluene. *Mol J Synthetic Chem Nat Prod Chem* **2017**, *22*, 564, doi:10.3390/molecules22040564.

11. Nevárez-Martínez, M.C.; Mazierski, P.; Kobylański, M.P.; Szczepańska, G.; Trykowski, G.; Malankowska, A.; Kozak, M.; Espinoza-Montero, P.J.; Zaleska-Medynska, A. Growth, Structure, and Photocatalytic Properties of Hierarchical V2O5–TiO2 Nanotube Arrays Obtained from the One-Step Anodic Oxidation of Ti–V Alloys. *Mol J Synthetic Chem Nat Prod Chem* **2017**, *22*, 580, doi:10.3390/molecules22040580.

12. Chirico, N.; Gramatica, P. Real External Predictivity of QSAR Models: How To Evaluate It? Comparison of Different Validation Criteria and Proposal of Using the Concordance Correlation Coefficient. *J Chem Inf Model* **2011**, *51*, 2320–2335, doi:10.1021/ci200211n.

13. Gramatica, P.; Chirico, N.; Papa, E.; Cassani, S.; Kovarich, S. QSARINS: A New Software for the Development, Analysis, and Validation of QSAR MLR Models. *J Comput Chem* **2013**, *34*, 2121–2132, doi:10.1002/jcc.23361.
